# Supplementary material for: Transformation of valence signaling in a striatopallidal circuit
Source: bioRxiv. 2023 Dec 11:2023.08.01.551547. Originally published 2023 Aug 3. Preprint. [Version 2] doi: 10.1101/2023.08.01.551547 (PMC10418236; doi:10.1101/2023.08.01.551547)
Supplement: Supplement 1 [file media-1.pdf]

Figure S1:

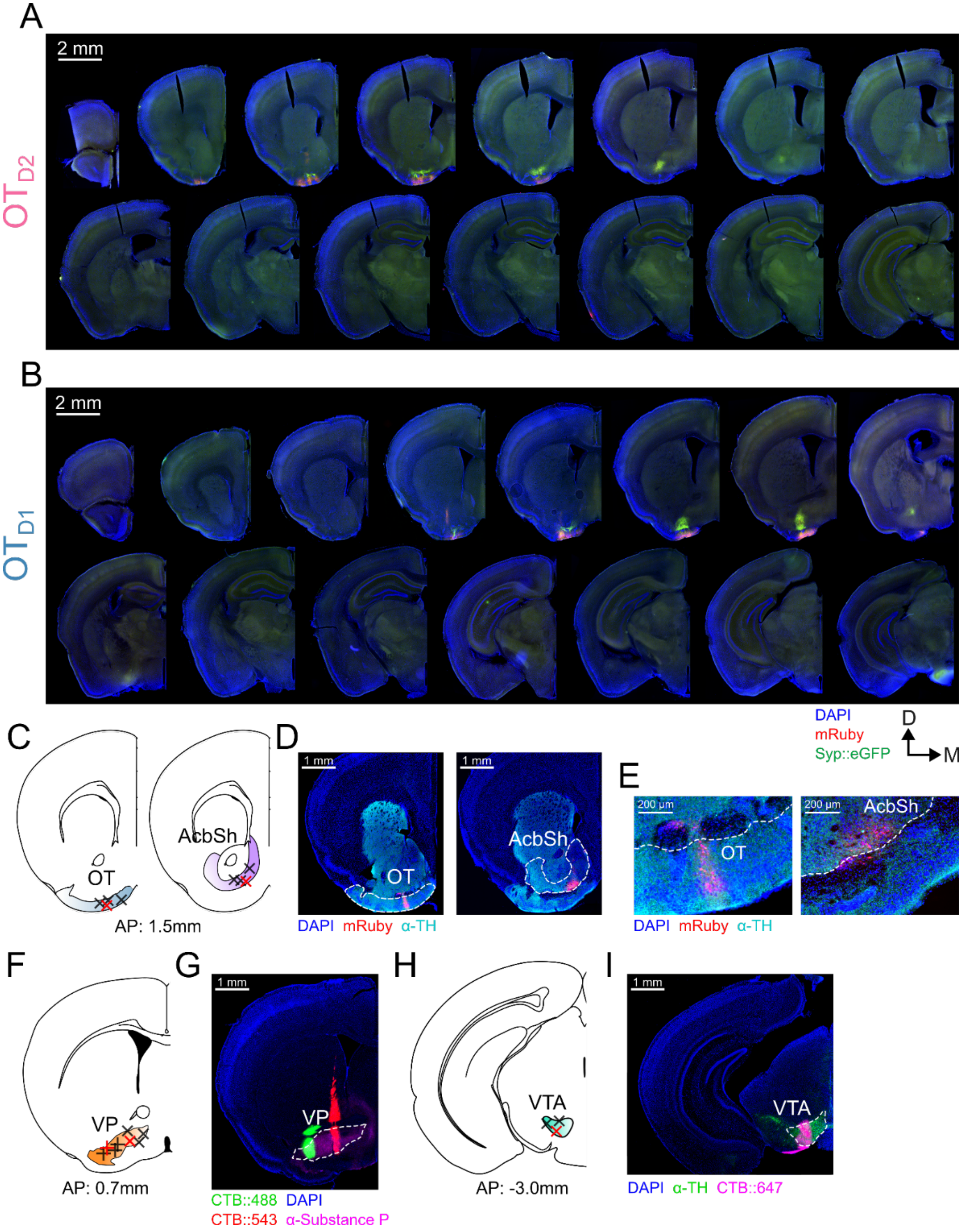
A
2 mm
OT D2
B
2 mm
OT D1
DAPI
mRuby
Syp::eGFP
D
M
C
AcbSh
OT
AP: 1.5mm
D
1 mm
OT
1 mm
AcbSh
DAPI mRuby α-TH
E
200 µm
OT
200 µm
AcbSh
DAPI mRuby α-TH
F
VP
AP: 0.7mm
G
1 mm
VP
CTB::488 DAPI
CTB::543 α-Substance P
H
VTA
AP: -3.0mm
I
1 mm
VTA
DAPI α-TH CTB::647

Figure S1. OT_{D1} and OT_{D2} primarily project to the lateral portion of the VP.
(**A**) Serial coronal sections from a representative experiment where AAVDJ-hSyn-FLEX-mRuby-T2A-syn-eGFP virus was injected into the anterior OT of an *Adora2a*-Cre mouse. Sections are roughly 400μm apart from each other and range from +2.5mm to -3.0mm relative to bregma. (**B**) Same as (A) but in a *Drd1*-Cre mouse. (**C**) Schematic showing the centroids of 4 injection sites for OT and AcbSh anterograde tracing experiments. (**D**, **E**) Representative images of the injection sites shown in (C). Sections were counterstained with α-TH to delineate the boundary between the striatum and the rostral ventral pallidum. The centroids of these samples are marked as red x's in (C). (**F**) Schematic showing the centroids of 4 CTB injection sites for lateral and medial VP. CTB::488 injection to the lateral VP are marked by +'s whereas CTB::543 injection to the medial VP are marked by x's. (**G**) Representative image of the injection sites shown in (F). Sections were counterstained with α-Substance P to mark the boundary of the VP. The centroids of this sample are marked by the red + and red x in (F). (**H**) Schematic showing the centroids of 3 CTB injection sites for VTA. (**I**) Representative image of the injection sites shown in (H). Sections were counterstained with α-TH to mark the boundary of the VTA.

Figure S2:

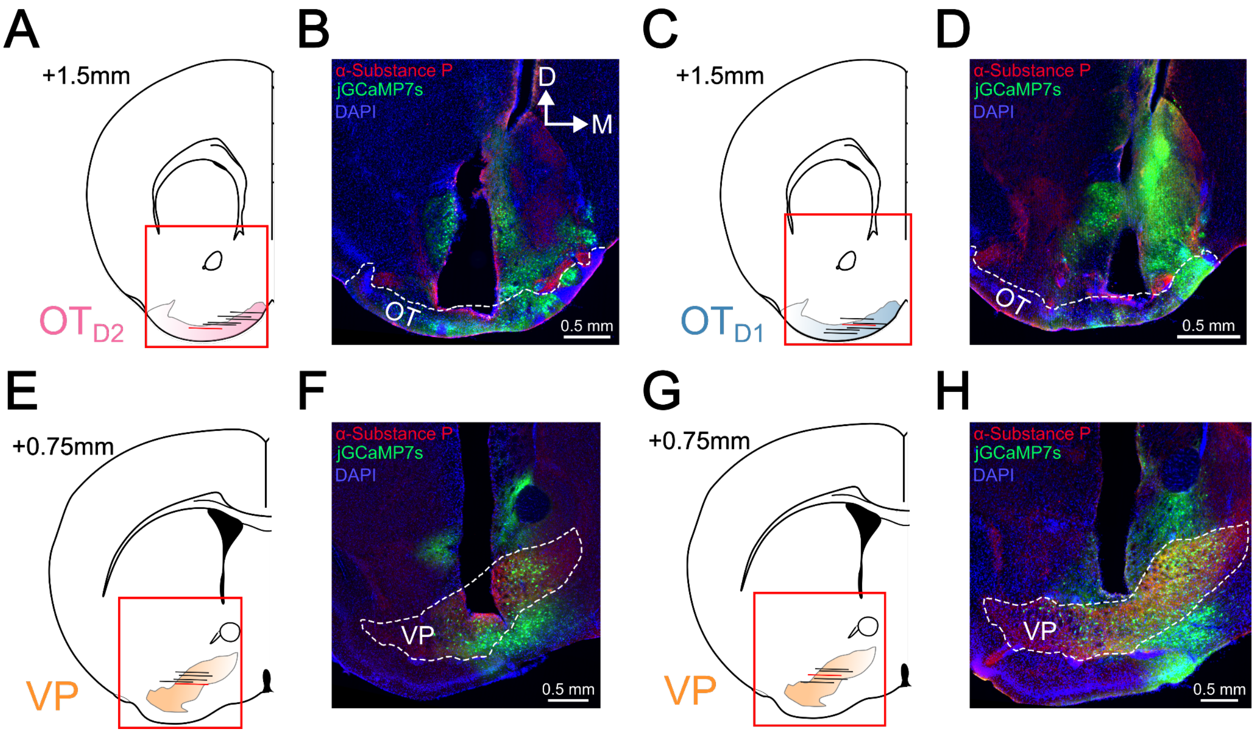

A
+1.5mm
OT D2
B
α-Substance P
jGCaMP7s
DAPI
D
M
OT
0.5 mm
C
+1.5mm
OT D1
D
α-Substance P
jGCaMP7s
DAPI
OT
0.5 mm
E
+0.75mm
VP
F
α-Substance P
jGCaMP7s
DAPI
VP
0.5 mm
G
+0.75mm
VP
H
α-Substance P
jGCaMP7s
DAPI
VP
0.5 mm

Figure S2. Histological verification of lens implant location. (**A**) Schematic showing the center, along the AP axis, of the implanted GRIN lens in 6 OT_{D2} jGCaMP7s animals. (**B**) Representative image of lens implant sites shown in (A). Sections were counterstained with α-Substance P to delineate the boundary of the VP. This sample is marked by a red horizontal line in (A). (**C**) Schematic as in (A) for 6 OT_{D1} jGCaMP7s animals. (**D**) Representative image of lens implant sites shown in (C). (**E**) Schematic as in (A) for 5 VP jGCaMP7s animals. (**F**) Representative image of lens implant sites shown in (E). (**G**) Schematic as in (A) for 5 VP jGCaMP7s animals recorded during the lick spout retraction paradigm (Figure 6). (**H**) Representative image of lens implant sites shown in (G). Sections were counterstained with α-Substance P to delineate the boundary of the VP. This sample is marked by a red horizontal line in (G).

Figure S3:

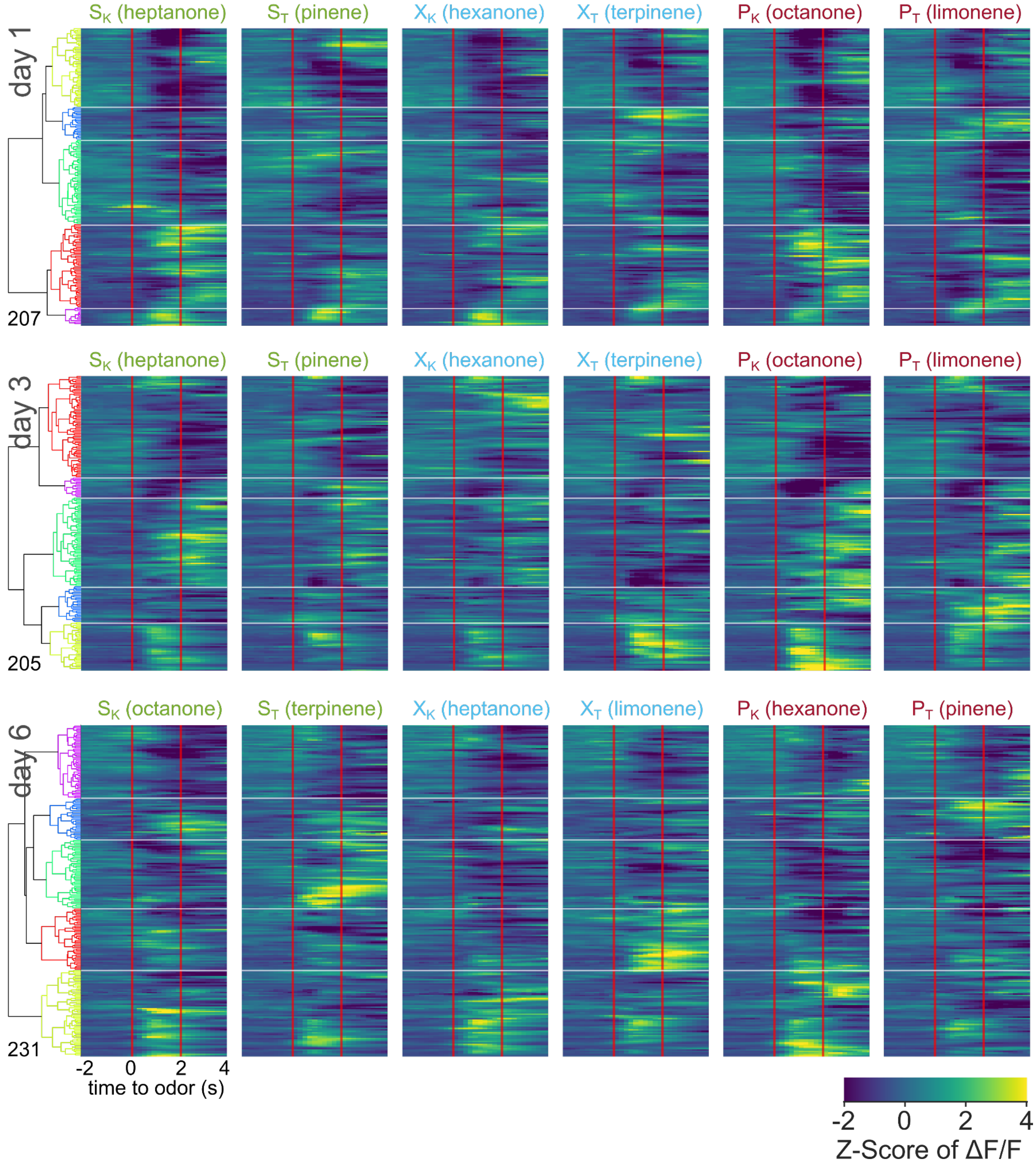

S_K (heptanone)
S_T (pinene)
X_K (hexanone)
X_T (terpinene)
P_K (octanone)
P_T (limonene)
day 1
207
day 3
205
S_K (octanone)
S_T (terpinene)
X_K (heptanone)
X_T (limonene)
P_K (hexanone)
P_T (pinene)
day 6
231
-2 0 2 4
time to odor (s)
-2 0 2 4
Z-Score of ΔF/F

Figure S3. Pooled averaged-over-trials neural activity of all neurons from OT_{D2} animals across days. Heatmap of odor-evoked activity in OT_{D2} neurons from day 1, day 3, and day 6 of imaging. The fluorescence measurements from each neuron were averaged over trials, Z-scored, then pooled for hierarchical clustering. Neurons are grouped by similarity, with the dendrogram shown on the right. Horizontal white lines demarcate the boundaries between the 6 clusters. Odor delivered at 0-2 seconds marked by vertical red lines. From left to right, the columns represent neural responses to sucrose-paired ketone and terpene, control ketone and terpene, and airpuff-paired ketone and terpene (S_K, S_T, X_K, X_T, P_K, P_T). Data is pooled from 6 animals.

Figure S4:

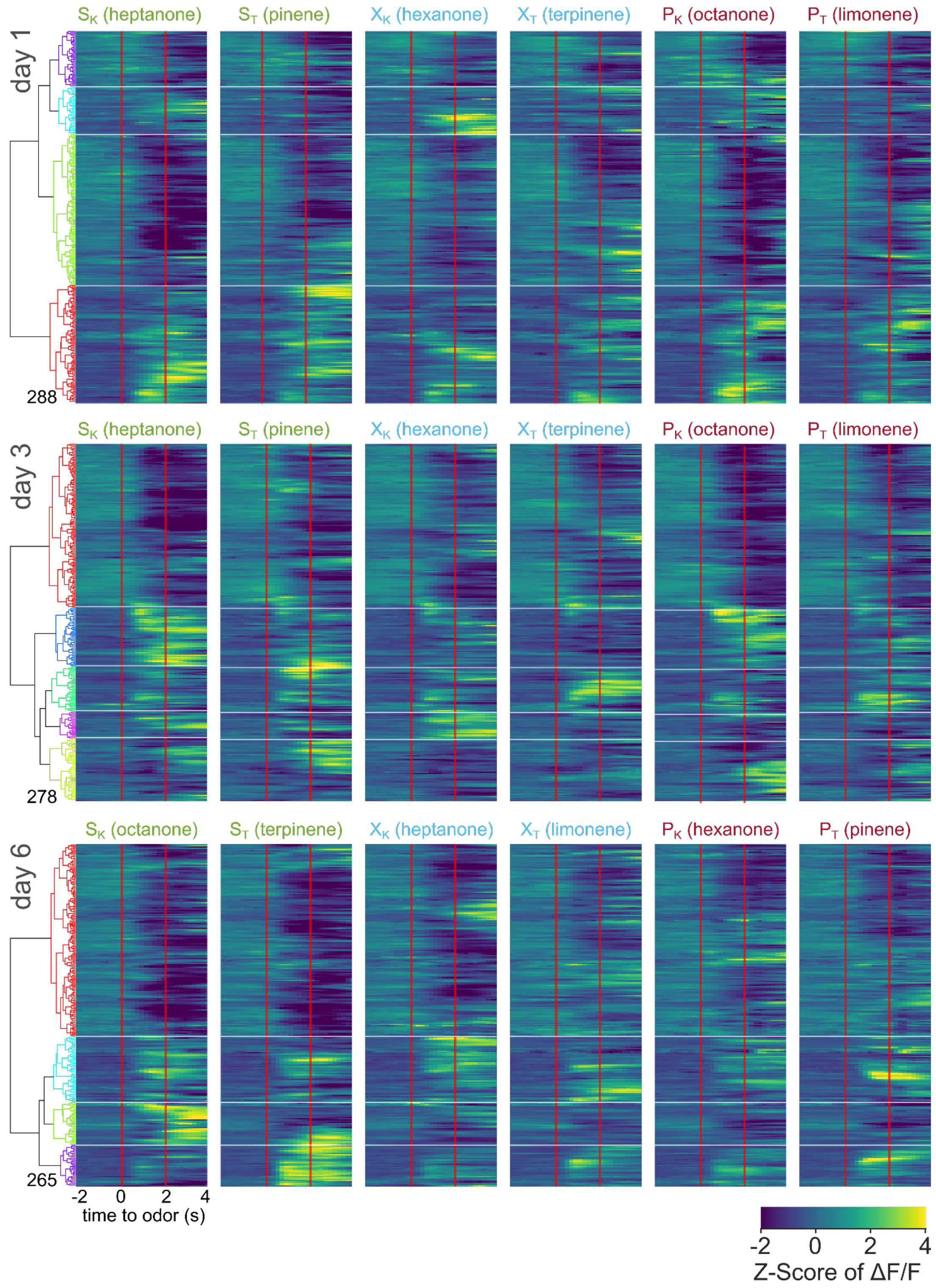

S_K (heptanone)
S_T (pinene)
X_K (hexanone)
X_T (terpinene)
P_K (octanone)
P_T (limonene)
day 1
288
day 3
278
S_K (octanone)
S_T (terpinene)
X_K (heptanone)
X_T (limonene)
P_K (hexanone)
P_T (pinene)
day 6
265
-2
0
2
4
time to odor (s)
-2
0
2
4
Z-Score of ΔF/F

Figure S4. Pooled averaged-over-trials neural activity of all neurons from OT_{D1} animals across days. Heatmap of odor-evoked activity in OT_{D1} neurons from day 1, day 3, and day 6 of imaging.. The fluorescence measurements from each neuron were averaged over trials, Z-scored, then pooled for hierarchical clustering. Neurons are grouped by similarity, with the dendrogram shown on the right. Horizontal white lines demarcate the boundaries between the 6 clusters. Odor delivered at 0-2 seconds marked by vertical red lines. From left to right, the columns represent neural responses to sucrose-paired ketone and terpene, control ketone and terpene, and airpuff-paired ketone and terpene (S_K, S_T, X_K, X_T, P_K, P_T). Data is pooled from 6 animals.

Figure S5:

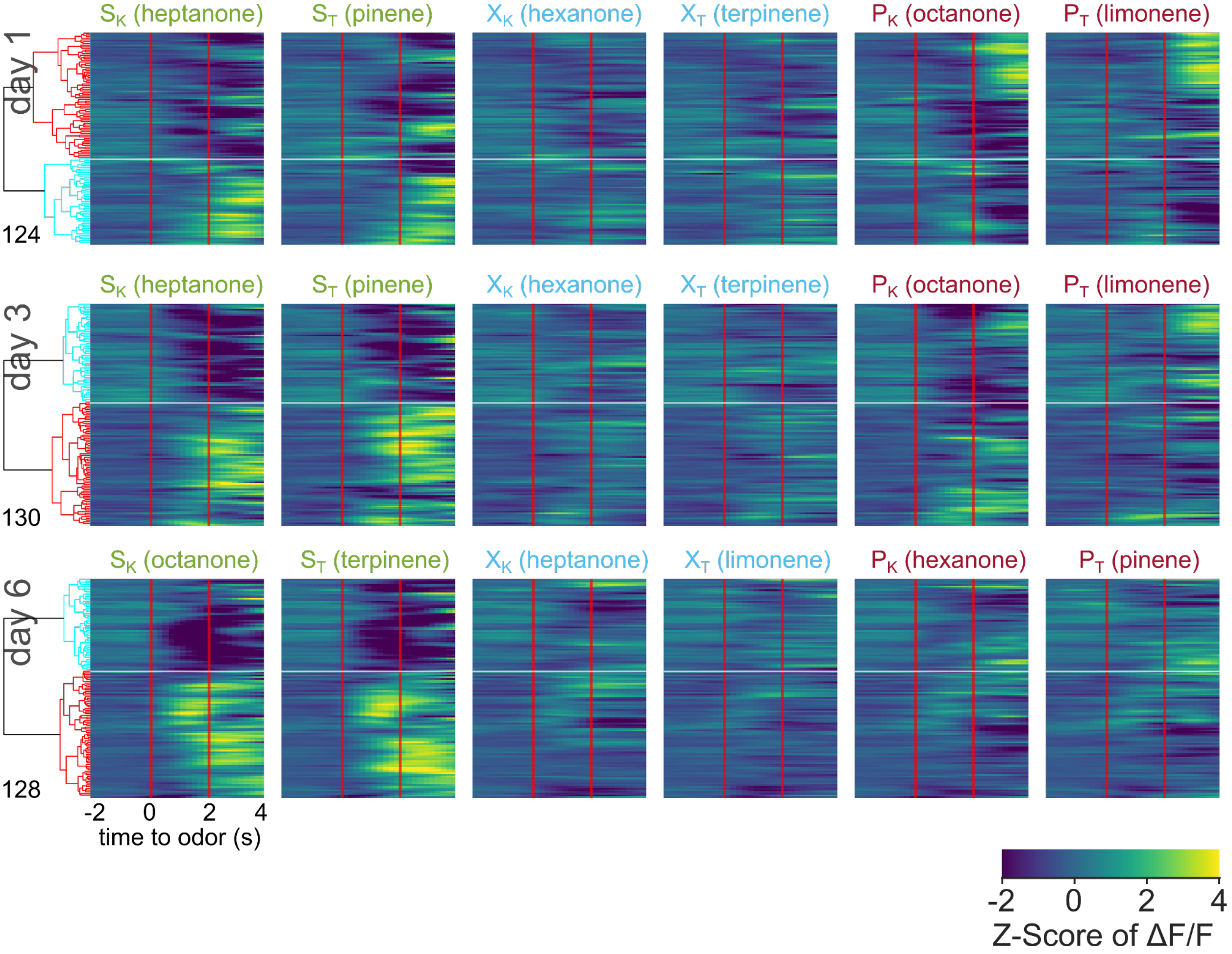

S_K (heptanone)
S_T (pinene)
X_K (hexanone)
X_T (terpinene)
P_K (octanone)
P_T (limonene)
day 1
124
S_K (heptanone)
S_T (pinene)
X_K (hexanone)
X_T (terpinene)
P_K (octanone)
P_T (limonene)
day 3
130
S_K (octanone)
S_T (terpinene)
X_K (heptanone)
X_T (limonene)
P_K (hexanone)
P_T (pinene)
day 6
128
-2
0
2
4
time to odor (s)
-2
0
2
4
Z-Score of ΔF/F

Figure S5. Pooled averaged-over-trials neural activity of all neurons from VP animals across days. Heatmap of odor-evoked activity in VP neurons from day 1, day 3, and day 6 of imaging. The fluorescence measurements from each neuron were averaged over trials, Z-scored, then pooled for hierarchical clustering. Neurons are grouped by similarity, with the dendrogram shown on the right. Horizontal white lines demarcate the boundaries between the 6 clusters. Odor delivered at 0-2 seconds marked by vertical red lines. From left to right, the columns represent neural responses to sucrose-paired ketone and terpene, control ketone and terpene, and airpuff-paired ketone and terpene (S_K, S_T, X_K, X_T, P_K, P_T). Data is pooled from 5 animals.

Figure S6:

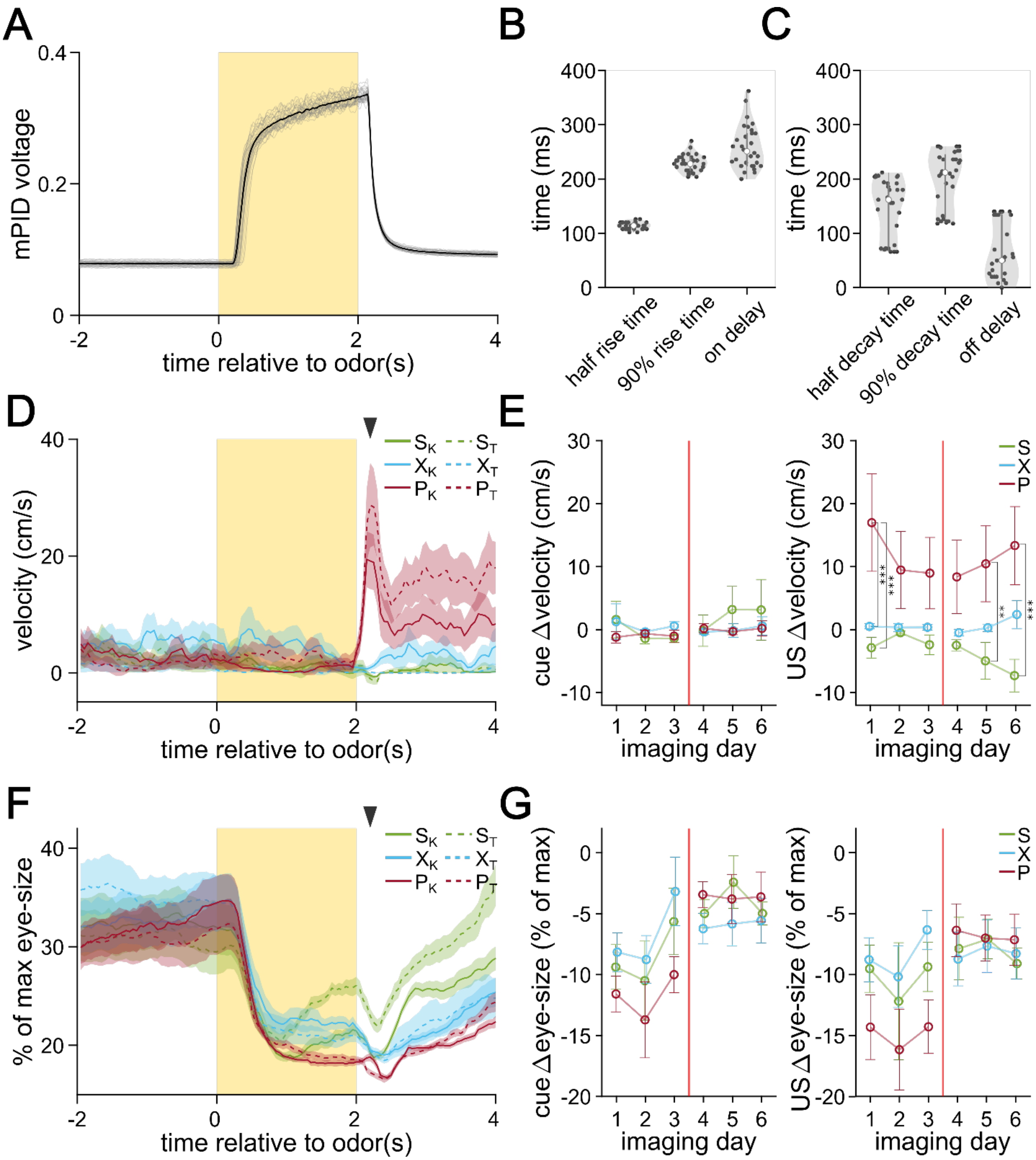

A
mPID voltage
time relative to odor(s)
B
time (ms)
half rise time
90% rise time
on delay
C
time (ms)
half decay time
90% decay time
off delay
D
velocity (cm/s)
time relative to odor(s)
E
cue Δvelocity (cm/s)
US Δvelocity (cm/s)
imaging day
F
% of max eye-size
time relative to odor(s)
G
cue Δeye-size (% of max)
US Δeye-size (% of max)
imaging day

Figure S6. Extended behavioral analysis from imaging period. (**A**) mPID voltage reading in response to 30 trials of a sample odor (α-terpinene) delivery. The time period during which the odor valve was turned on is shown by the yellow rectangle. Individual recordings are shown in gray and the average is shown in black. (**B**) On-kinetics of odor delivery. On delay refers to the interval between the valve turning on and the mPID voltage increasing by more than 10% of baseline. (**C**) Off-kinetics of odor delivery. Off delay refers to the interval between the odor valve turning off and the mPID voltage decreasing by more than 10% of its maximum. (**D**) Representative velocity of the head-fixed mouse in response to the 6 different odors measured by a digital encoder. The lines represent the average across 30 trials and the shaded areas represent the SEM. The black arrowhead marks when the US is delivered. (**E**) The difference in walking velocity in response to odor (left) and US delivery (right). Differences are calculated between the last second before odor delivery and the last second before the odor exposure (left) or the first half second after US delivery (right) grouped by US pairing. Circles represent the average across animals and the error bars show SEM. (**F**) Representative changes in range-normalized eye-size in response to the 6 different odors. (**G**) The difference in eye-size in response to odor (left) and US delivery (right). Differences are calculated between the last second before odor delivery and the last second before the odor exposure (left) or the first half second after US delivery (right) grouped by US pairing. Circles represent the average across animals and the error bars show SEM. FWER-adjusted statistical significance for post hoc comparisons are shown as: ***$p<0.001$, **$p<0.01$, *$p<0.05$, n.s. $p>0.05$. See Tables S32-36 for detailed statistics.

Figure S7:

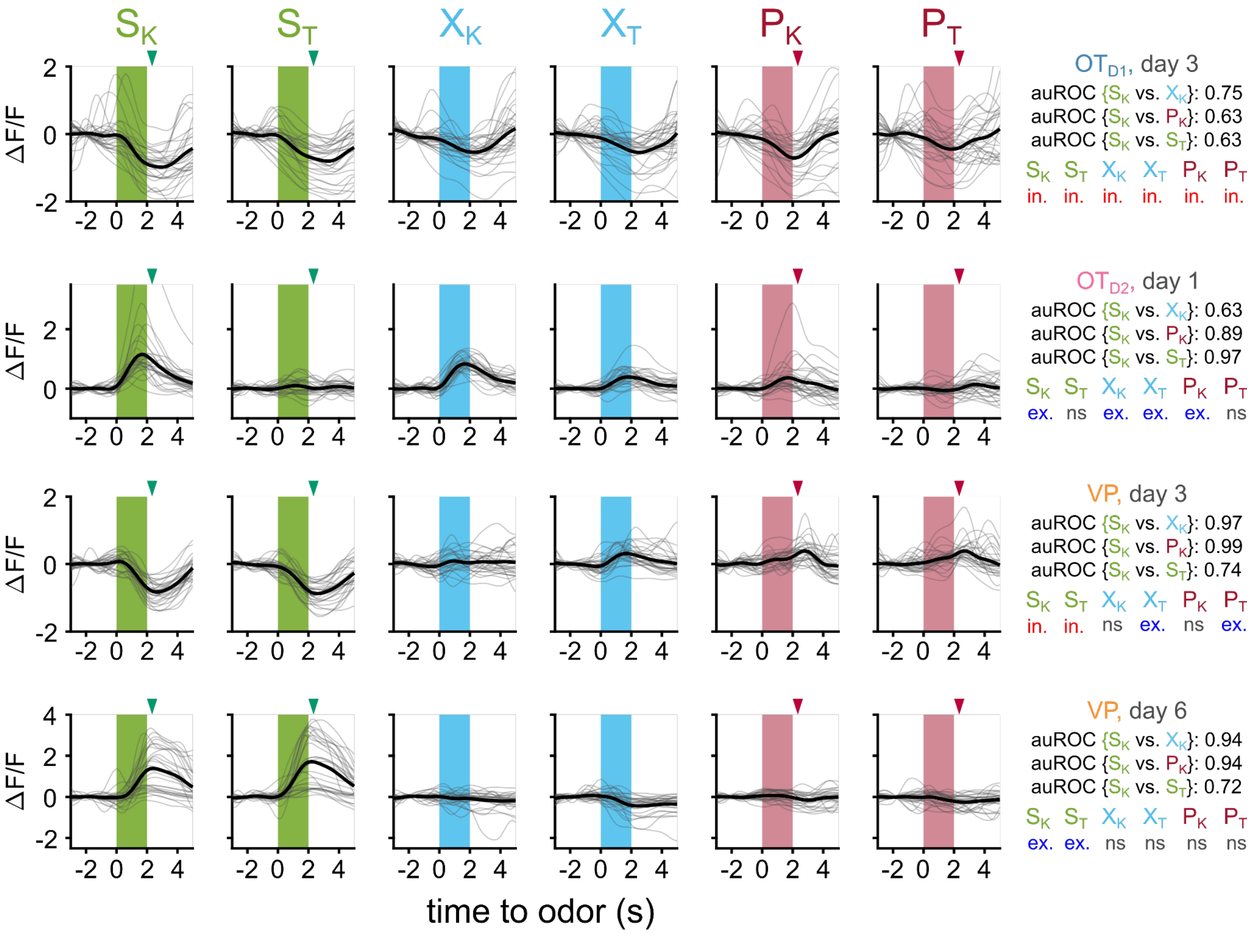

S_K S_T X_K X_T P_K P_T
ΔF/F
time to odor (s)
OT_D1, day 3
auROC {S_K vs. X_K}: 0.75
auROC {S_K vs. P_K}: 0.63
auROC {S_K vs. S_T}: 0.63
S_K S_T X_K X_T P_K P_T
in. in. in. in. in. in.
OT_D2, day 1
auROC {S_K vs. X_K}: 0.63
auROC {S_K vs. P_K}: 0.89
auROC {S_K vs. S_T}: 0.97
S_K S_T X_K X_T P_K P_T
ex. ns ex. ex. ex. ns
VP, day 3
auROC {S_K vs. X_K}: 0.97
auROC {S_K vs. P_K}: 0.99
auROC {S_K vs. S_T}: 0.74
S_K S_T X_K X_T P_K P_T
in. in. ns ex. ns ex.
VP, day 6
auROC {S_K vs. X_K}: 0.94
auROC {S_K vs. P_K}: 0.94
auROC {S_K vs. S_T}: 0.72
S_K S_T X_K X_T P_K P_T
ex. ex. ns ns ns ns

Figure S7. Traces of example neurons and their corresponding metrics. (**A**) Example traces from an OT_{D1} neuron recorded on day 3. Each column shows this neuron's response to a given odor across 30 trials (gray). The average across all trials is shown in black. For green and red arrowheads mark the time at which sucrose and airpuff were delivered, respectively. auROC values from single-neuron binary classifiers for discriminating {S_K vs. X_K}, {S_K vs. P_K}, and {S_K vs. S_T} are displayed on the right. Additionally, the results of statistical analysis to determine if this neuron reliably responded to each odor (in. = significant inhibitory response, exc. = significant excitatory response, ns= no significant difference between baseline and odor period). (**B**) Example traces from an OT_{D2} neuron recorded on day 1. (**C**) Example traces from a VP neuron recorded on day 3. (**D**) Example traces from a VP neuron recorded on day 6.

Figure S8:

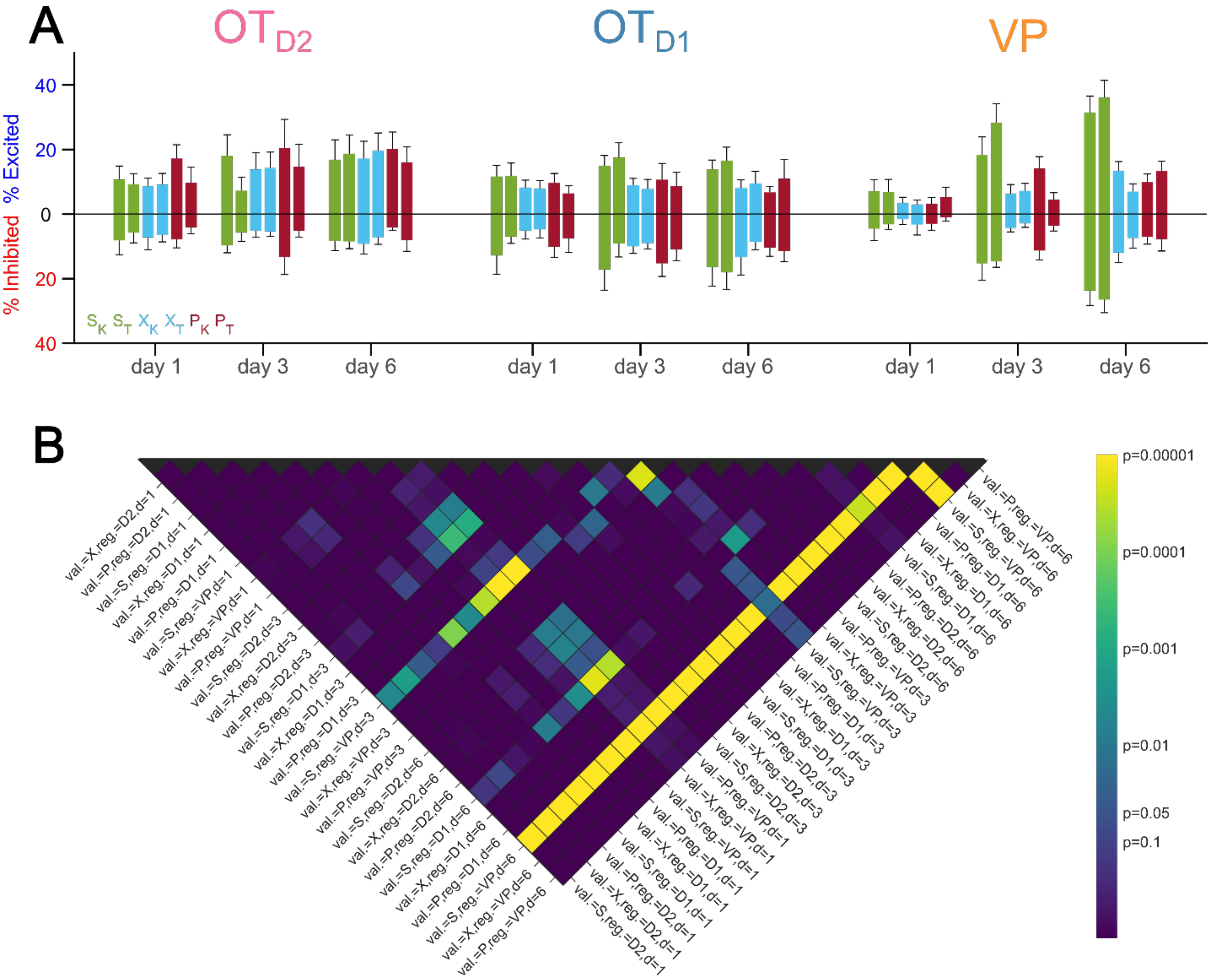

A
OTD2
OTD1
VP
% Inhibited % Excited
40
20
0
20
40
SK ST XK XT PK PT
day 1
day 3
day 6
B
p=0.00001
p=0.0001
p=0.001
p=0.01
p=0.05
p=0.1

Figure S8. Percentage of neurons responsive to each odor across days. (**A**) Bar graphs showing percentage of neurons from each region on imaging days 1, 3 and 6 that were significantly excited or inhibited by each odor. The average across animals is shown by the bar and the error bars represent SEM. (**B**) Heatmap of post hoc pairwise comparison p-values of percent responsive across imaging days and imaging region. See Table S37 for detailed statistics.

Figure S9:

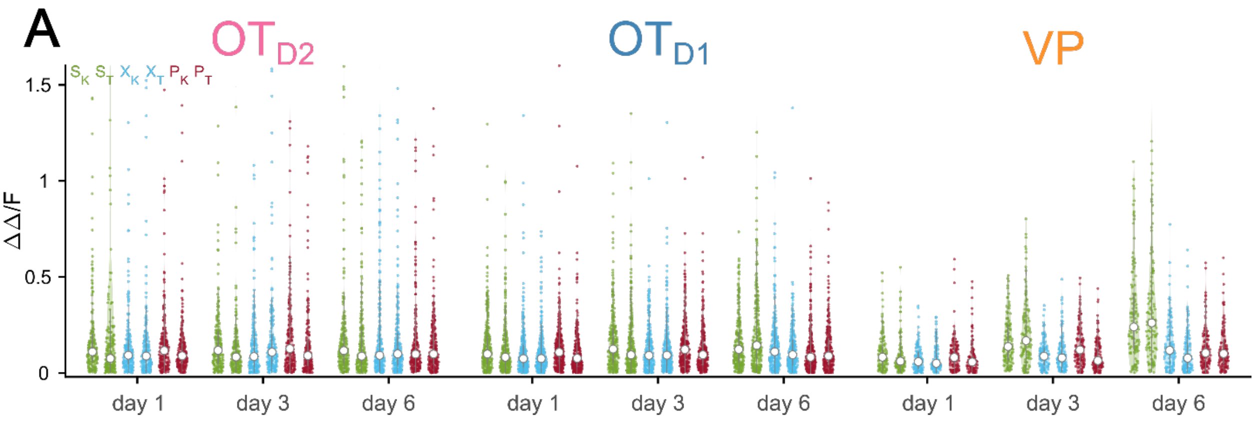
A
OT$_{D2}$
OT$_{D1}$
VP
S$_K$ S$_T$ X$_K$ X$_T$ P$_K$ P$_T$
ΔΔ/F
1.5
1
0.5
0
day 1
day 3
day 6
day 1
day 3
day 6
day 1
day 3
day 6

Figure S9. Distribution of response magnitudes to each odor across days. Violin plots showing the averaged-over-trials response magnitudes to each odor during the last second of odor exposure. See Table S38 for detailed statistics.

Figure S10:

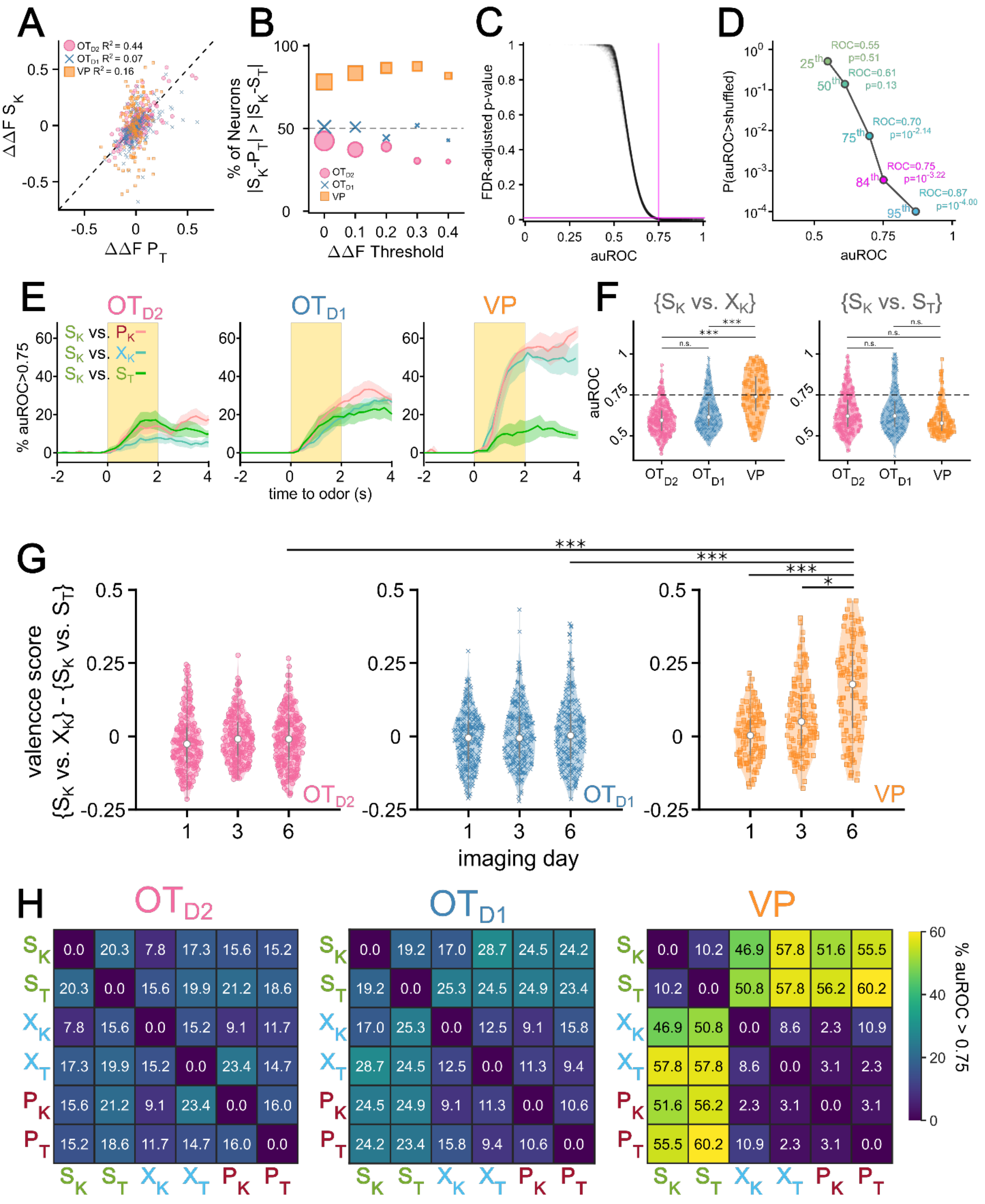

A
OTD2 R2 = 0.44
OTD1 R2 = 0.07
VP R2 = 0.16
ΔΔF SK
ΔΔF PT
B
% of Neurons |SK-PT| > |SK-ST|
ΔΔF Threshold
C
FDR-adjusted p-value
auROC
D
P(auROC>shuffled)
ROC=0.55 p=0.51
ROC=0.61 p=0.13
ROC=0.70 p=10-2.14
ROC=0.75 p=10-3.22
ROC=0.87 p=10-4.00
25th
50th
75th
84th
95th
auROC
E
OTD2
OTD1
VP
SK vs. PK
SK vs. XK
SK vs. ST
% auROC>0.75
time to odor (s)
F
{SK vs. XK}
{SK vs. ST}
auROC
n.s.
***
G
valencce score
{SK vs. XK} - {SK vs. ST}
imaging day
H
OTD2
OTD1
VP
% auROC > 0.75
SK ST XK XT PK PT
0.0 20.3 7.8 17.3 15.6 15.2
20.3 0.0 15.6 19.9 21.2 18.6
7.8 15.6 0.0 15.2 9.1 11.7
17.3 19.9 15.2 0.0 23.4 14.7
15.6 21.2 9.1 23.4 0.0 16.0
15.2 18.6 11.7 14.7 16.0 0.0
0.0 19.2 17.0 28.7 24.5 24.2
19.2 0.0 25.3 24.5 24.9 23.4
17.0 25.3 0.0 12.5 9.1 15.8
28.7 24.5 12.5 0.0 11.3 9.4
24.5 24.9 9.1 11.3 0.0 10.6
24.2 23.4 15.8 9.4 10.6 0.0
0.0 10.2 46.9 57.8 51.6 55.5
10.2 0.0 50.8 57.8 56.2 60.2
46.9 50.8 0.0 8.6 2.3 10.9
57.8 57.8 8.6 0.0 3.1 2.3
51.6 56.2 2.3 3.1 0.0 3.1
55.5 60.2 10.9 2.3 3.1 0.0

Figure S10. Pairwise analysis of single neuron odor encoding.

(**A**) Scatterplot comparing the magnitudes of S_K responses ($\Delta\Delta S_K$) to P_T responses ($\Delta\Delta P_T$). The dotted line represents the hypothetical scenario where $\Delta\Delta S_K = \Delta\Delta P_T$. For each population, the R^2 value of the 2-d distribution compared to the $\Delta\Delta S_K = \Delta\Delta P_T$ line is reported. (**B**) The percentage of neurons from each population where the difference between $\Delta\Delta S_K$ and $\Delta\Delta P_T$ is lower than that between $\Delta\Delta S_K$ and $\Delta\Delta S_T$. (**C**) Bootstrapped FDR-adjusted p-values as a function of auROC values of single-neuron binary classifiers. In total, there are 27,900 single-neuron binary classifiers (15 pairwise classifiers for each of the 1860 recordings across 3 regions and days 1, 3 and 6 of imaging). Each classifier was compared against 10,000 shuffles. Horizontal magenta line marks FDR-adjusted p-value of 0.001 and the vertical magenta line marks auROC of 0.75. (**D**) The 25th, 50th, 75th, 84th and 95th percentiles of auROC values and their corresponding unadjusted p-values. For auROC values that were greater than all 10,000 shuffles, a conservative p-value of 0.0001 was assigned. (**E**) The percentage of day 6 {S_K vs P_K}, {S_K vs X_K}, and {S_K vs S_T} auROC values greater than 0.75 as a function of time relative to odor, grouped by region. Lines represent the average across biological replicates and the shaded area shows the SEM. (**F**) Violin plot showing the distribution of pooled day 6 {S_K vs X_K} (left) and {S_K vs S_T} (right) auROC values grouped by region. Horizontal dotted line marks auROC = 0.75. (**G**) Violin plot of the distribution of single-neuron valence scores (defined as the difference between the average auROC for {S vs. X|P} classification and {S_K vs. S_T} classification), grouped by imaging day and region. (**H**) Heatmap of percentage of single-neuron pairwise classifiers with auROC>0.75. Classifiers were trained from neural activity recorded during the last second of odor exposure. Percentage of neurons with auROC>0.75 for a given binary classification was averaged across animals and grouped by region. For post hoc pairwise comparisons, the median values for all neurons in each animal were compared across imaging day and region. The FWER-adjusted p-values are shown as: ***p<0.001, **p<0.01, *p<0.05, n.s. p>0.05. See Tables S39-46 for detailed statistics.

Figure S11:

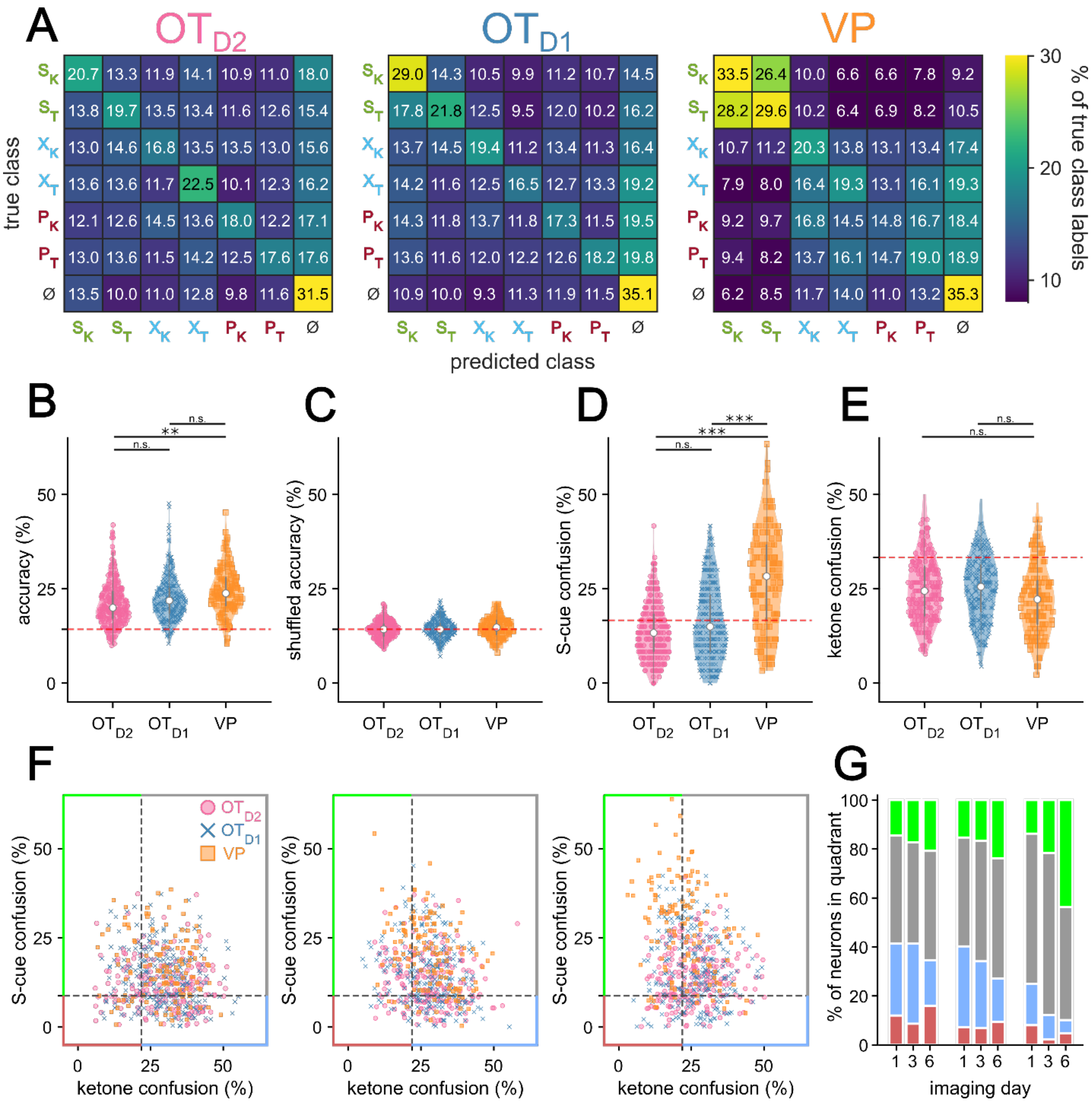
A
OTD2
OTD1
VP
true class
predicted class
% of true class labels
30
20
10
SK ST XK XT PK PT Ø
20.7 13.3 11.9 14.1 10.9 11.0 18.0
13.8 19.7 13.5 13.4 11.6 12.6 15.4
13.0 14.6 16.8 13.5 13.5 13.0 15.6
13.6 13.6 11.7 22.5 10.1 12.3 16.2
12.1 12.6 14.5 13.6 18.0 12.2 17.1
13.0 13.6 11.5 14.2 12.5 17.6 17.6
13.5 10.0 11.0 12.8 9.8 11.6 31.5
29.0 14.3 10.5 9.9 11.2 10.7 14.5
17.8 21.8 12.5 9.5 12.0 10.2 16.2
13.7 14.5 19.4 11.2 13.4 11.3 16.4
14.2 11.6 12.5 16.5 12.7 13.3 19.2
14.3 11.8 13.7 11.8 17.3 11.5 19.5
13.6 11.6 12.0 12.2 12.6 18.2 19.8
10.9 10.0 9.3 11.3 11.9 11.5 35.1
33.5 26.4 10.0 6.6 6.6 7.8 9.2
28.2 29.6 10.2 6.4 6.9 8.2 10.5
10.7 11.2 20.3 13.8 13.1 13.4 17.4
7.9 8.0 16.4 19.3 13.1 16.1 19.3
9.2 9.7 16.8 14.5 14.8 16.7 18.4
9.4 8.2 13.7 16.1 14.7 19.0 18.9
6.2 8.5 11.7 14.0 11.0 13.2 35.3
B
C
D
E
accuracy (%)
shuffled accuracy (%)
S-cue confusion (%)
ketone confusion (%)
n.s.
**
***
OTD2 OTD1 VP
0
25
50
F
OTD2
OTD1
VP
S-cue confusion (%)
ketone confusion (%)
G
% of neurons in quadrant
imaging day
1 3 6
0
20
40
60
80
100

Figure S11. Multinomial analysis of single neuron odor encoding (**A**) Confusion matrix of single-neuron MNR classifiers trained on neural activity during the last second of odor exposure on day 6 of imaging. Rows represent the true class while columns represent the predicted class. Each confusion matrix is averaged across 10 k-fold and across all neurons of a given region. ϕ represents data taken from 30 pre-odor bins randomly sampled from -1.5 to -0.5 seconds relative to odor delivery. (**B**) Violin plot of single-neuron MNR classifier accuracy, averaged across 10 k-fold, grouped by region. (**C**) Violin plot of the single-neuron MNR classifier accuracy trained on shuffled data. Each data point represents the average across 10 shuffles. (**D**) Violin plot of MNR S-cue confusion, i.e. confusion between S_K and S_T. This corresponds to 1) when the true class was S_K but predicted class was S_T and 2) when the true class was S_T but the predicted class was S_K. (**E**) Violin plot of MNR confusion among all ketones. This corresponds when the true class was a ketone and the predicted class was a different ketone (e.g. true class = X_K and predicted class = P_K). (**F**) Scatterplot of each neuron's ketone confusion on the x-axis and S-cue confusion on the y-axis on days 1, 3, and 6 of imaging. (**G**) Stacked bar graph showing the distribution of neurons from each population that fall into each of the 4 quadrants across the 3 different imaging days. For post hoc pairwise comparisons, the median values for all neurons in each animal were compared across imaging day and region. The FWER-adjusted p-values are shown as: ***p<0.001, **p<0.01, *p<0.05, n.s. p>0.05. See Tables S47-53 for detailed statistics.

Figure S12:

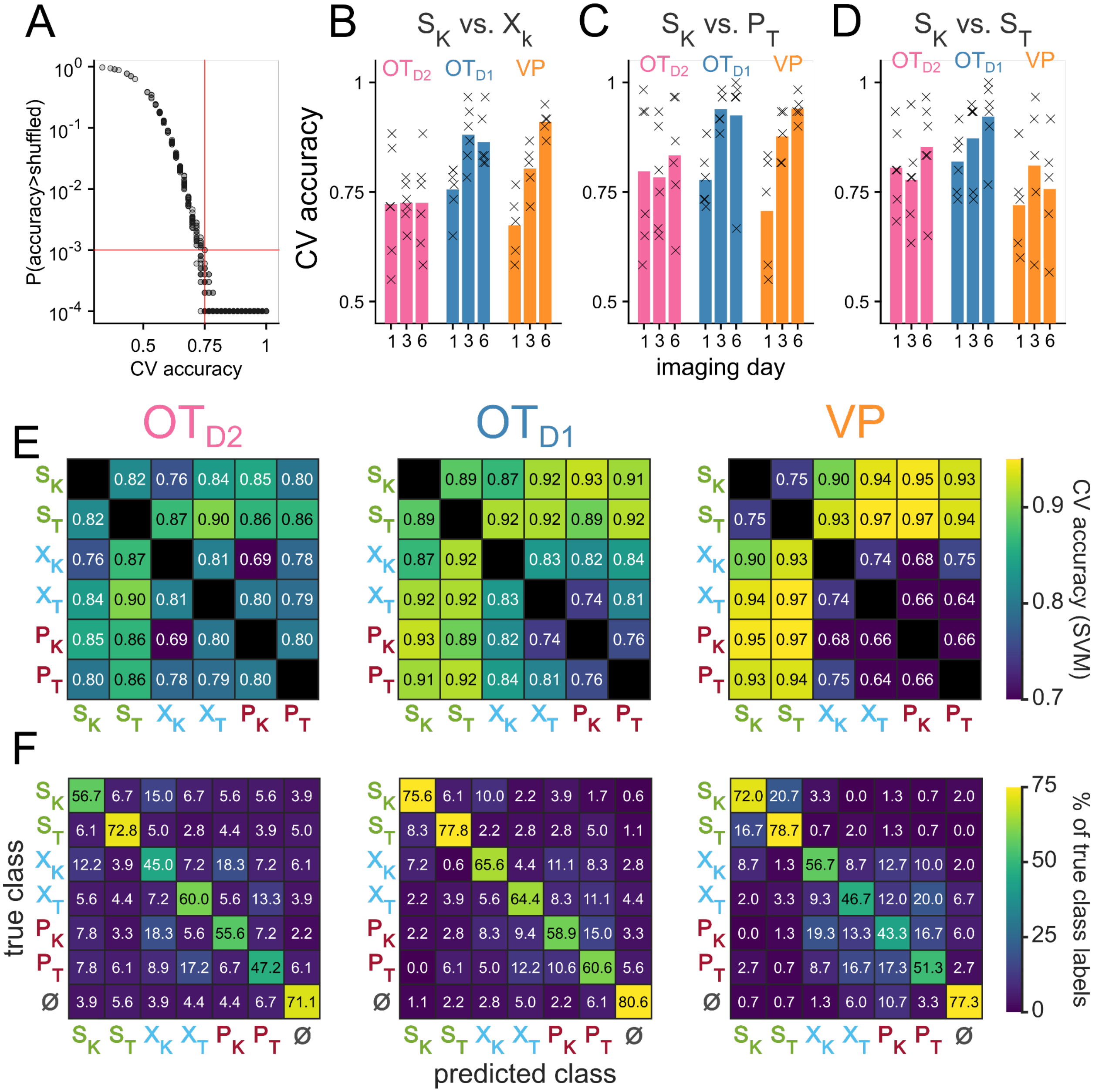
A
P(accuracy>shuffled)
10^0 10^{-1} 10^{-2} 10^{-3} 10^{-4}
0.5 0.75 1
CV accuracy
B
S_K vs. X_k
C
S_K vs. P_T
D
S_K vs. S_T
OT_{D2} OT_{D1} VP
CV accuracy
1 0.75 0.5
1 3 6 1 3 6 1 3 6
imaging day
E
OT_{D2}
OT_{D1}
VP
S_K S_T X_K X_T P_K P_T
0.82 0.76 0.84 0.85 0.80
0.82 0.87 0.90 0.86 0.86
0.76 0.87 0.81 0.69 0.78
0.84 0.90 0.81 0.80 0.79
0.85 0.86 0.69 0.80 0.80
0.80 0.86 0.78 0.79 0.80
0.89 0.87 0.92 0.93 0.91
0.89 0.92 0.92 0.89 0.92
0.87 0.92 0.83 0.82 0.84
0.92 0.92 0.83 0.74 0.81
0.93 0.89 0.82 0.74 0.76
0.91 0.92 0.84 0.81 0.76
0.75 0.90 0.94 0.95 0.93
0.75 0.93 0.97 0.97 0.94
0.90 0.93 0.74 0.68 0.75
0.94 0.97 0.74 0.66 0.64
0.95 0.97 0.68 0.66 0.66
0.93 0.94 0.75 0.64 0.66
CV accuracy (SVM)
0.9 0.8 0.7
F
true class
S_K S_T X_K X_T P_K P_T Ø
56.7 6.7 15.0 6.7 5.6 5.6 3.9
6.1 72.8 5.0 2.8 4.4 3.9 5.0
12.2 3.9 45.0 7.2 18.3 7.2 6.1
5.6 4.4 7.2 60.0 5.6 13.3 3.9
7.8 3.3 18.3 5.6 55.6 7.2 2.2
7.8 6.1 8.9 17.2 6.7 47.2 6.1
3.9 5.6 3.9 4.4 4.4 6.7 71.1
75.6 6.1 10.0 2.2 3.9 1.7 0.6
8.3 77.8 2.2 2.8 2.8 5.0 1.1
7.2 0.6 65.6 4.4 11.1 8.3 2.8
2.2 3.9 5.6 64.4 8.3 11.1 4.4
2.2 2.8 8.3 9.4 58.9 15.0 3.3
0.0 6.1 5.0 12.2 10.6 60.6 5.6
1.1 2.2 2.8 5.0 2.2 6.1 80.6
72.0 20.7 3.3 0.0 1.3 0.7 2.0
16.7 78.7 0.7 2.0 1.3 0.7 0.0
8.7 1.3 56.7 8.7 12.7 10.0 2.0
2.0 3.3 9.3 46.7 12.0 20.0 6.7
0.0 1.3 19.3 13.3 43.3 16.7 6.0
2.7 0.7 8.7 16.7 17.3 51.3 2.7
0.7 0.7 1.3 6.0 10.7 3.3 77.3
predicted class
% of true class labels
75 50 25 0

Figure S12. Analysis of population-level odor encoding (**A**) Scatterplot of CV-accuracy of linear classifiers trained on simultaneously-recorded neurons on the x-axis and their bootstrapped unadjusted p-values on the y-axis. Red horizontal line marks p = 0.001 and red red vertical line marks CV accuracy = 0.75. All classifiers with CV accuracy higher than 0.75 had $p<0.001$. In total, there are 765 binary classifiers (15 pairwise classifiers for each of the 51 recordings across 3 regions and days 1, 3, and 6 of imaging). Each classifier was compared against 10,000 shuffles. For auROC values that were greater than all 10,000 shuffles, a conservative p-value of 0.0001 was assigned. (**B**) The CV accuracy for {S_K vs X_K} binary classification trained on the last second of population-level activity. The bars represent the average across biological replicates. CV accuracy from individual animals are shown as x's. (**C**) Same as (B) but for {S_K vs. P_T} classification. (**D**) Same as (B) but for {S_K vs. S_T} classification. (**E**) Heatmap of CV accuracy from binary SVM's trained on day 6 of imaging with a radial basis function kernel. CV accuracy was averaged across biological replicates. (**F**) Confusion matrix of population-level MNR classifiers trained on neural activity during the last second of odor exposure on day 6 of imaging. Rows represent the true class while columns represent the predicted class. Each confusion matrix is averaged across biological replicates. ϕ represents data taken from 30 pre-odor bins randomly sampled from -1.5 to -0.5 seconds relative to odor delivery. See Tables S54-59 for details on statistical comparison of average classifier accuracy across animals.

Figure S13:

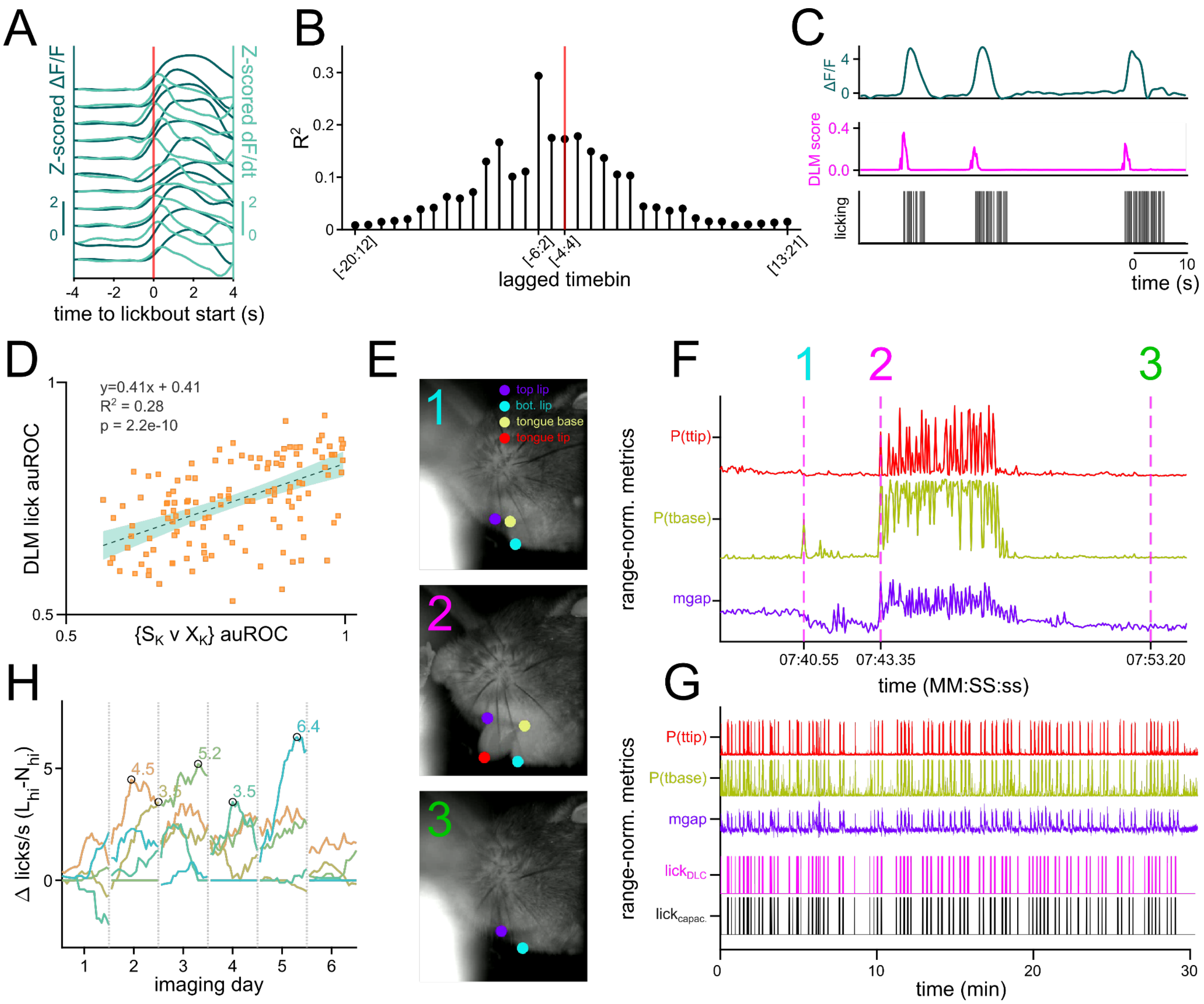
A
Z-scored ΔF/F
Z-scored dF/dt
2
0
-4 -2 0 2 4
time to lickbout start (s)
B
R²
0.3
0.2
0.1
0
[-20:12]
[-6:2]
[-4:4]
[13:21]
lagged timebin
C
ΔF/F
4
0
DLM score
0.4
0.0
licking
0 10
time (s)
D
y=0.41x + 0.41
R² = 0.28
p = 2.2e-10
DLM lick auROC
{S_K v X_K} auROC
0.5
1
E
1
top lip
bot. lip
tongue base
tongue tip
2
3
F
1
2
3
range-norm. metrics
P(ttip)
P(tbase)
mgap
07:40.55
07:43.35
07:53.20
time (MM:SS:ss)
G
range-norm. metrics
P(ttip)
P(tbase)
mgap
lick_DLC
lick_capac.
0 10 20 30
time (min)
H
Δ licks/s (L_hi-N_hi)
5
0
4.5
3.5
5.2
3.5
6.4
1 2 3 4 5 6
imaging day

Figure S13. Camera-based detection of licking in head-fixed animals (**A-C**) Metrics of a representative neurons with activity that predicts licking. (**A**) Representative neuron's Z-scored ΔF/F (dark green) and Z-scored dF/dt (light green) aligned to the onset of a lickbout. Each line represents the same neuron's activity during an individual lickbout. (**B**) Stemplot showing an example of the lagged correlation between the onset of licking and the fluorescence of a neuron across 9 frames (1 frame = 0.2s). Time bins of various lags are shown on the x-axis (negative number denotes frames that precede onset of licking) and the resulting R^2 is plotted on the y-axis. Red vertical line marks the case where the 9 frames are centered on the onset of licking. As an example, [-6:2] refers to fluorescence between 1.2 seconds prior to lickbout onset and 0.4 seconds after lickbout onset. (**C**) The output of a distributed lag model (DLM) that predicts the onset of a lickbout from ΔF/F of a single neuron. ΔF/F (dark green, top), DLM score (magenta, middle), and the licking recorded by a capacitive sensor (black, bottom) are shown in parallel. The DLM model was trained using 9 distributed frames ([-6:2]) of ΔF/F for each frame of lickbout onset. (**D**) Scatterplot of day 6 VP neurons' DLM lick classifier auROC on the y-axis plotted against their mean {S_K vs. X_K} auROC on the x-axis. The slope, intercept, R^2, and p-value of the slope are shown on the top left corner. (**E**) 3 example snapshots of the camera feed during moving lick spout paradigm with overlay of DeepLabCut labeling. The coordinates of top lip, bottom lip, base of tongue (tbase), and tip of tongue (ttip) are displayed with a probability cutoff of 0.4. (**F**) Range-normalized metrics from DLC labeling. P(ttip) (red, top) is the probability score assigned to the labeling of the tongue tip. P(tbase) (yellow, middle) is the probability score assigned to the labeling of the tongue base. Mgap (purple, bottom) is the Euclidean distance between the top lip and the bottom lip. The 3 vertical magenta lines represent the timing of the 3 snapshots shown in (E). (**G**) The same range-normalized metrics as in (F) plotted against the ground truth licking data from capacitive sensor (black, bottom) and DLC-based licking classifier score (magenta, second from bottom). (**H**) Lineplot showing the difference in total licking to L_{hi} and N_{hi} during the time bin (1.5-2.5 seconds after odor onset) used for most analyses plotted against imaging day for individual animals. The time of peak difference is circled in black.

Supplemental Tables

Table S1:

**Group A**	**Group B**	**Lower Limit**	**A-B**	**Upper Limit**	**FDR adjusted P-value**
OT_{D1} → VP	Acb_{D1} → VP	-20.506	-8.798	2.910	0.577
OT_{D1} → VP	OT_{D2} → VP	-0.851	9.922	20.694	0.577
OT_{D1} → VP	Acb_{D2} → VP	9.338	19.340	29.341	0.291
Acb_{D1} → VP	OT_{D2} → VP	11.163	18.719	26.275	0.161
Acb_{D1} → VP	Acb_{D2} → VP	21.729	28.137	34.545	3.394E-02
OT_{D2} → VP	Acb_{D2} → VP	4.942	9.418	13.894	0.206
OT_{D1} → VP	Acb_{D1} → VP	-9.944	-8.199	-6.453	2.223E-02
OT_{D1} → LH	OT_{D2} → LH	-0.045	0.564	1.173	0.577
OT_{D1} → LH	Acb_{D2} → LH	-0.016	0.591	1.198	0.577
Acb_{D1} → LH	OT_{D2} → LH	7.125	8.763	10.401	2.223E-02
Acb_{D1} → LH	Acb_{D2} → LH	7.153	8.790	10.426	2.223E-02
OT_{D2} → LH	Acb_{D2} → LH	-0.029	0.027	0.082	0.655
OT_{D1} → VTA	Acb_{D1} → VTA	-17.357	-14.504	-11.650	2.223E-02
OT_{D1} → VTA	OT_{D2} → VTA	-0.022	0.183	0.387	0.577

Table S1. Pairwise comparisons of anterograde labeling from OT and AcbSh (Fig1C).

Table S2:

Group A	Group B	Lower Limit	A-B	Upper Limit	FDR adjusted P-value
AI→vlVP	AI→dmVP	78.249	148.500	218.751	0.116
Acb→vlVP	Acb→dmVP	-185.929	-123.250	-60.571	0.116
LS→vlVP	LS→dmVP	-130.496	-118.750	-107.004	3.27E-04
OFC→vlVP	OFC→dmVP	-167.021	-132.000	-96.979	1.86E-02
OT→vlVP	OT→dmVP	179.793	221.750	263.707	5.57E-03
Pir→vlVP	Pir→dmVP	-71.946	-21.500	28.946	0.685

Table S2. Pairwise comparisons of retrograde labeling from vlVP and dmVP (Fig1F).

Table S3:

Group A	Group B	Lower Limit	A-B	Upper Limit	FDR adjusted P-value
OT→VTA	AcbSh→VTA	-735.101	-575	-414.899	3.44E-02
OT→VTA	AcbC→VTA	-1027.381	-915	-802.619	3.71E-03
AcbC→VTA	AcbSh→VTA	144.452	340	535.548	0.157

Table S3. Pairwise comparisons of retrograde labeling from VTA (Fig1I).

Table S4:

**Source**	**Sum Sq.**	**d.f.**	**Mean Sq.**	**F**	**Prob>F**
day	4.301	5	0.860	27.638	2.29E-16
region	0.143	2	0.072	2.301	0.106
day:region	0.251	10	0.025	0.806	0.623
Error	2.583	83	0.031		
Total	7.305	100			

Table S4. 2-way ANOVA for effect of day or lens placement on licking accuracy (Fig2H).

Table S5:

Group A	Group B	Lower Limit	A-B	Upper Limit	P-value
day 1	day 2	-0.385	-0.208	-0.031	1.18E-02
day 1	day 3	-0.632	-0.452	-0.272	2.03E-09
day 1	day 4	-0.134	0.043	0.220	0.980
day 1	day 5	-0.558	-0.381	-0.203	2.30E-07
day 1	day 6	-0.650	-0.473	-0.295	2.58E-10
day 2	day 3	-0.424	-0.244	-0.064	2.18E-03
day 2	day 4	0.074	0.251	0.429	1.14E-03
day 2	day 5	-0.350	-0.172	0.005	0.061
day 2	day 6	-0.441	-0.264	-0.087	5.33E-04
day 3	day 4	0.315	0.495	0.675	8.31E-11
day 3	day 5	-0.109	0.071	0.251	0.856
day 3	day 6	-0.200	-0.021	0.159	0.999
day 4	day 5	-0.601	-0.424	-0.247	1.00E-08
day 4	day 6	-0.693	-0.516	-0.338	9.77E-12
day 5	day 6	-0.269	-0.092	0.085	0.657

Table S5. Post hoc pairwise comparisons of licking accuracy across imaging days (Fig2H).

Table S6:

**Source**	**Sum Sq.**	**d.f.**	**Mean Sq.**	**F**	**Prob>F**
day	0.05917	2	0.02958	2.99563	0.06079
region	0.03696	2	0.01848	1.87142	0.16651
day:region	0.06481	4	0.01620	1.64062	0.18197
Error	0.41479	42	0.00988		
Total	0.57170	50			

Table S6. 2-way ANOVA for effect of day or lens placement on percentage of neurons responsive to a single odor (Fig3E).

Table S7:

Group A	Group B	Lower Limit	A-B	Upper Limit	P-value
d1,D2 OT	d3,D2 OT	-0.140	0.048	0.236	0.995
d1,D2 OT	d6,D2 OT	-0.164	0.024	0.211	1.000
d3,D2 OT	d6,D2 OT	-0.212	-0.024	0.163	1.000
d1,D1 OT	d3,D1 OT	-0.287	-0.099	0.088	0.724
d1,D1 OT	d6,D1 OT	-0.328	-0.141	0.047	0.285
d3,D1 OT	d6,D1 OT	-0.229	-0.041	0.146	0.998
d1,VP	d3,VP	-0.321	-0.115	0.090	0.662
d1,VP	d6,VP	-0.335	-0.129	0.076	0.513
d3,VP	d6,VP	-0.220	-0.014	0.191	1.000
d1,D2 OT	d1,D1 OT	-0.115	0.072	0.260	0.937
d1,D2 OT	d1,VP	-0.056	0.141	0.338	0.341
d1,D1 OT	d1,VP	-0.128	0.069	0.265	0.964
d3,D2 OT	d3,D1 OT	-0.263	-0.075	0.112	0.923
d3,D2 OT	d3,VP	-0.219	-0.022	0.175	1.000
d3,D1 OT	d3,VP	-0.144	0.053	0.250	0.993
d6,D2 OT	d6,D1 OT	-0.280	-0.092	0.095	0.796
d6,D2 OT	d6,VP	-0.209	-0.012	0.184	1.000
d6,D1 OT	d6,VP	-0.117	0.080	0.276	0.918

Table S7. Post hoc pairwise comparisons of percentage of neurons responsive to a single odor across imaging days and region (Fig3E).

Table S8:

**Source**	**Sum Sq.**	**d.f.**	**Mean Sq.**	**F**	**Prob>F**
day	0.10897	2	0.05448	4.58607	0.01580
region	0.00469	2	0.00234	0.19732	0.82168
day:region	0.06640	4	0.01660	1.39730	0.25144
Error	0.49897	42	0.01188		
Total	0.66598	50			

Table S8. 2-way ANOVA for effect of day or lens placement on percentage of neurons responsive to 3 or more odors (Fig3E).

Table S9:

Group A	Group B	Lower Limit	A-B	Upper Limit	P-value
d1,D2 OT	d3,D2 OT	-0.237	-0.031	0.175	1.000
d1,D2 OT	d6,D2 OT	-0.246	-0.040	0.165	0.999
d3,D2 OT	d6,D2 OT	-0.215	-0.009	0.196	1.000
d1,D1 OT	d3,D1 OT	-0.230	-0.024	0.182	1.000
d1,D1 OT	d6,D1 OT	-0.269	-0.063	0.143	0.984
d3,D1 OT	d6,D1 OT	-0.245	-0.039	0.167	0.999
d1,VP	d3,VP	-0.406	-0.181	0.044	0.207
d1,VP	d6,VP	-0.453	-0.228	-0.003	0.046
d3,VP	d6,VP	-0.272	-0.047	0.178	0.999
d1,D2 OT	d1,D1 OT	-0.220	-0.014	0.191	1.000
d1,D2 OT	d1,VP	-0.124	0.092	0.308	0.894
d1,D1 OT	d1,VP	-0.109	0.106	0.322	0.793
d3,D2 OT	d3,D1 OT	-0.213	-0.007	0.198	1.000
d3,D2 OT	d3,VP	-0.274	-0.058	0.158	0.993
d3,D1 OT	d3,VP	-0.266	-0.051	0.165	0.997
d6,D2 OT	d6,D1 OT	-0.243	-0.037	0.168	1.000
d6,D2 OT	d6,VP	-0.311	-0.096	0.120	0.871
d6,D1 OT	d6,VP	-0.274	-0.059	0.157	0.993

Table S9. Post hoc pairwise comparisons of percentage of neurons responsive to 3 or more odors across imaging days and region (Fig3E).

Table S10:

Source	Sum Sq.	d.f.	Mean Sq.	F	Prob>F
day	0.11460	2	0.05730	6.60475	0.00321
region	0.18370	2	0.09185	10.58706	0.00019
day:region	0.16522	4	0.04131	4.76096	0.00294
Error	0.36438	42	0.00868		
Total	0.80767	50			

Table S10. 2-way ANOVA for effect of day or lens placement on percentage of neurons responsive to both S-cues (Fig3E).

Table S11:

Group A	Group B	Lower Limit	A-B	Upper Limit	P-value
d1,D2 OT	d3,D2 OT	-0.139	0.037	0.213	0.999
d1,D2 OT	d6,D2 OT	-0.200	-0.024	0.151	1.000
d3,D2 OT	d6,D2 OT	-0.237	-0.062	0.114	0.963
d1,D1 OT	d3,D1 OT	-0.239	-0.063	0.112	0.957
d1,D1 OT	d6,D1 OT	-0.196	-0.020	0.156	1.000
d3,D1 OT	d6,D1 OT	-0.133	0.043	0.219	0.996
d1,VP	d3,VP	-0.441	-0.248	-0.056	3.79E-03
d1,VP	d6,VP	-0.473	-0.281	-0.088	7.12E-04
d3,VP	d6,VP	-0.225	-0.032	0.160	1.000
d1,D2 OT	d1,D1 OT	-0.189	-0.013	0.163	1.000
d1,D2 OT	d1,VP	-0.151	0.033	0.217	1.000
d1,D1 OT	d1,VP	-0.138	0.046	0.230	0.996
d3,D2 OT	d3,D1 OT	-0.289	-0.114	0.062	0.480
d3,D2 OT	d3,VP	-0.437	-0.252	-0.068	1.72E-03
d3,D1 OT	d3,VP	-0.323	-0.139	0.045	0.279
d6,D2 OT	d6,D1 OT	-0.185	-0.009	0.167	1.000
d6,D2 OT	d6,VP	-0.408	-0.223	-0.039	7.94E-03
d6,D1 OT	d6,VP	-0.399	-0.214	-0.030	1.23E-02

Table S11. Post hoc pairwise comparisons of percentage of neurons responsive to both S-cues across imaging days and region (Fig3E).

Table S12:

**Source**	**Sum Sq.**	**d.f.**	**Mean Sq.**	**F**	**Prob>F**
day	0.85429	2	0.42714	46.23443	2.439E-11
region	0.32224	2	0.16112	17.43954	3.064E-06
day:region	0.40111	4	0.10028	10.85411	3.918E-06
Error	0.38802	42	0.00924		
Total	1.87808	50			

Table S12. 2-way ANOVA for effect of day or lens placement on percentage of neurons with auROC>0.75 for {S_K vs. P_K} (Fig3l).

Table S13:

Group A	Group B	Lower Limit	A-B	Upper Limit	P-value
d1,D2 OT	d3,D2 OT	-0.259	-0.078	0.103	0.890
d1,D2 OT	d6,D2 OT	-0.279	-0.097	0.084	0.709
d3,D2 OT	d6,D2 OT	-0.201	-0.020	0.162	1.000
d1,D1 OT	d3,D1 OT	-0.293	-0.112	0.069	0.541
d1,D1 OT	d6,D1 OT	-0.417	-0.236	-0.054	0.003
d3,D1 OT	d6,D1 OT	-0.305	-0.124	0.058	0.407
d1,VP	d3,VP	-0.476	-0.278	-0.079	0.001
d1,VP	d6,VP	-0.820	-0.621	-0.423	2.00E-11
d3,VP	d6,VP	-0.542	-0.344	-0.145	4.09E-05
d1,D2 OT	d1,D1 OT	-0.170	0.011	0.192	1.000
d1,D2 OT	d1,VP	-0.141	0.049	0.239	0.995
d1,D1 OT	d1,VP	-0.152	0.038	0.228	0.999
d3,D2 OT	d3,D1 OT	-0.204	-0.023	0.158	1.000
d3,D2 OT	d3,VP	-0.341	-0.151	0.039	0.221
d3,D1 OT	d3,VP	-0.318	-0.128	0.062	0.427
d6,D2 OT	d6,D1 OT	-0.309	-0.127	0.054	0.370
d6,D2 OT	d6,VP	-0.665	-0.475	-0.285	1.15E-08
d6,D1 OT	d6,VP	-0.538	-0.348	-0.158	1.43E-05

Table S13. Post hoc comparisons of percentage of neurons with auROC>0.75 for {S_K vs. P_K} across imaging day and region (Fig3l).

Table S14:

**Source**	**Sum Sq.**	**d.f.**	**Mean Sq.**	**F**	**Prob>F**
day	0.551	2	0.275	28.099	1.794E-08
region	0.377	2	0.188	19.265	1.156E-06
day:region	0.364	4	0.0912	9.310	1.764E-05
Error	0.411	42	0.009		
Total	1.637	50			

Table S14. 2-way ANOVA for effect of day or lens placement on percentage of neurons with auROC>0.75 for {S_K vs. X_K} (Fig3J).

Table S15:

Group A	Group B	Lower Limit	A-B	Upper Limit	P-value
d1,D2 OT	d3,D2 OT	-0.189	-0.002	0.184	1.000
d1,D2 OT	d6,D2 OT	-0.238	-0.052	0.135	0.992
d3,D2 OT	d6,D2 OT	-0.236	-0.049	0.137	0.994
d1,D1 OT	d3,D1 OT	-0.313	-0.126	0.061	0.421
d1,D1 OT	d6,D1 OT	-0.356	-0.170	0.017	0.102
d3,D1 OT	d6,D1 OT	-0.230	-0.044	0.143	0.997
d1,VP	d3,VP	-0.454	-0.250	-0.045	7.23E-03
d1,VP	d6,VP	-0.750	-0.545	-0.341	2.05E-09
d3,VP	d6,VP	-0.500	-0.295	-0.091	8.14E-04
d1,D2 OT	d1,D1 OT	-0.219	-0.033	0.154	1.000
d1,D2 OT	d1,VP	-0.163	0.033	0.229	1.000
d1,D1 OT	d1,VP	-0.130	0.066	0.261	0.972
d3,D2 OT	d3,D1 OT	-0.343	-0.156	0.031	0.167
d3,D2 OT	d3,VP	-0.410	-0.214	-0.019	2.26E-02
d3,D1 OT	d3,VP	-0.254	-0.058	0.138	0.987
d6,D2 OT	d6,D1 OT	-0.337	-0.151	0.036	0.204
d6,D2 OT	d6,VP	-0.656	-0.461	-0.265	5.39E-08
d6,D1 OT	d6,VP	-0.506	-0.310	-0.114	1.95E-04

Table S15. Post hoc comparisons of percentage of neurons with auROC>0.75 for {S_K vs. X_K} across imaging day and region (Fig3J).

Table S16:

**Source**	**Sum Sq.**	**d.f.**	**Mean Sq.**	**F**	**Prob>F**
day	0.01400	2	0.00700	0.45251	0.63909
region	0.08031	2	0.04015	2.59595	0.08650
day:region	0.01282	4	0.00321	0.20726	0.93297
Error	0.64967	42	0.01547		
Total	0.75527	50			

Table S16. 2-way ANOVA for effect of day or lens placement on percentage of neurons with auROC>0.75 for {S_K vs. S_T} (Fig3K).

Table S17:

Group A	Group B	Lower Limit	A-B	Upper Limit	P-value
d1,D2 OT	d3,D2 OT	-0.237	-0.002	0.232	1.000
d1,D2 OT	d6,D2 OT	-0.276	-0.041	0.193	1.000
d3,D2 OT	d6,D2 OT	-0.274	-0.039	0.196	1.000
d1,D1 OT	d3,D1 OT	-0.255	-0.020	0.214	1.000
d1,D1 OT	d6,D1 OT	-0.233	0.001	0.236	1.000
d3,D1 OT	d6,D1 OT	-0.213	0.022	0.257	1.000
d1,VP	d3,VP	-0.316	-0.059	0.198	0.998
d1,VP	d6,VP	-0.336	-0.079	0.178	0.983
d3,VP	d6,VP	-0.277	-0.020	0.237	1.000
d1,D2 OT	d1,D1 OT	-0.267	-0.032	0.202	1.000
d1,D2 OT	d1,VP	-0.142	0.104	0.350	0.900
d1,D1 OT	d1,VP	-0.110	0.136	0.382	0.678
d3,D2 OT	d3,D1 OT	-0.285	-0.050	0.184	0.999
d3,D2 OT	d3,VP	-0.199	0.047	0.293	0.999
d3,D1 OT	d3,VP	-0.149	0.097	0.344	0.928
d6,D2 OT	d6,D1 OT	-0.224	0.011	0.245	1.000
d6,D2 OT	d6,VP	-0.180	0.066	0.312	0.993
d6,D1 OT	d6,VP	-0.191	0.055	0.302	0.998

Table S17. Post hoc comparisons of percentage of neurons with auROC>0.75 for {S_K vs. S_T} across imaging day and region (Fig3K).

Table S18:

**Group A**	**Group B**	**Lower Limit**	**A-B**	**Upper Limit**	**FDR adjusted P-value**
D2 OT	D1 OT	-0.204	-0.138	-0.073	4.37E-02
D2 OT	VP	-0.275	-0.214	-0.153	1.08E-03
D1 OT	VP	-0.120	-0.075	-0.031	0.141

Table S18. Pairwise comparisons of $|\Delta\Delta F_{day3}|-|\Delta\Delta F_{day1}|$ across regions (Fig4I).

Table S19:

Population	Lower Limit	Mean	Upper Limit	FDR adjusted P-value
D2 OT	-0.146	-6.51E-02	1.58E-02	0.259
D1 OT	2.78E-02	7.83E-02	0.129	4.48E-02
VP	0.133	0.174	0.215	2.49E-07

Table S19. One sample t-tests of $|\Delta\Delta F_{day3}|-|\Delta\Delta F_{day1}|$ in different regions (Fig4I).

Table S20:

Source	Sum Sq.	d.f.	Mean Sq.	F	Prob>F
region	0.018	2	9.10E-03	9.569	2.40E-03
Error	0.013	14	9.51E-04		
Total	0.032	16			

Table S20. One-way ANOVA for effect of region on {S vs. X|P} linear classifier accuracy (Fig5G).

Table S21:

**Group A**	**Group B**	**Lower Limit**	**A-B**	**Upper Limit**	**P-value**
D2 OT	D1 OT	-0.114	-0.067	-0.020	5.57E-03
D2 OT	VP	-0.119	-0.070	-0.021	5.65E-03
D1 OT	VP	-0.052	-0.003	0.046	0.985

Table S21. Post hoc comparisons of {S vs. X|P} linear classifier accuracy across regions (Fig5G).

Table S22:

Source	Sum Sq.	d.f.	Mean Sq.	F	Prob>F
region	0.134	2	0.067	14.136	4.37E-04
Error	0.066	14	0.005		
Total	0.201	16			

Table S22. One-way ANOVA for effect of region on generalized {S vs. X|P} linear classifier accuracy (Fig5G).

Table S23:

Group A	Group B	Lower Limit	A-B	Upper Limit	P-value
D2 OT	D1 OT	-0.153	-0.049	0.055	0.451
D2 OT	VP	-0.323	-0.214	-0.105	4.17E-04
D1 OT	VP	-0.274	-0.165	-0.056	3.84E-03

Table S23. Post hoc comparisons of generalized {S vs. X|P} linear classifier accuracy across regions (Fig5G).

Table S24:

**Source**	**Sum Sq.**	**d.f.**	**Mean Sq.**	**F**	**Prob>F**
days	0.775	2	0.387	0.277	0.759
region	50.226	2	25.113	17.969	2.704E-06
days:region	14.484	4	3.621	2.591	0.051

Table S24. 2-way ANOVA for effect of imaging days or region on normalized PR (Fig5l).

Table S25:

Group A	Group B	Lower Limit	A-B	Upper Limit	P-value
d1,D2 OT	d3,D2 OT	-2.938	-0.592	1.754	0.995
d1,D2 OT	d6,D2 OT	-3.700	-1.355	0.991	0.623
d3,D2 OT	d6,D2 OT	-2.999	-0.762	1.474	0.968
d1,D1 OT	d3,D1 OT	-1.948	0.289	2.526	1
d1,D1 OT	d6,D1 OT	-1.965	0.272	2.509	1
d3,D1 OT	d6,D1 OT	-2.254	-0.017	2.220	1
d1,VP	d3,VP	-2.404	0.195	2.794	1
d1,VP	d6,VP	-0.783	1.816	4.415	0.372
d3,VP	d6,VP	-0.829	1.621	4.071	0.445
d1,D2 OT	d3,D1 OT	-3.316	-0.971	1.375	0.907
d1,D2 OT	d1,VP	-1.921	0.678	3.277	0.994
d1,D1 OT	d1,VP	-0.563	1.938	4.438	0.245
d3,D2 OT	d3,D1 OT	-2.615	-0.378	1.858	1
d3,D2 OT	d3,VP	-0.881	1.465	3.811	0.522
d3,D1 OT	d3,VP	-0.502	1.843	4.189	0.229
d6,D2 OT	d6,D1 OT	-1.870	0.367	2.604	1
d6,D2 OT	d6,VP	1.503	3.848	6.194	1.14E-04
d6,D1 OT	d6,VP	1.135	3.481	5.827	5.67E-04

Table S25. Post hoc comparisons of normalized PR across imaging day and region (Fig5I).

Table S26:

**Source**	**Sum Sq.**	**d.f.**	**Mean Sq.**	**F**	**Prob>F**
region	0.209	2	0.104	20.965	6.16E-05
Error	0.070	14	0.005		
Total	0.279	16			

Table S26. One-way ANOVA for effect of region on {S_K vs. P_K} linear classifier accuracy trained on PC1 (Fig5L).

Table S27:

Group A	Group B	Lower Limit	A-B	Upper Limit	P-value
D2 OT	D1 OT	-0.172	-0.066	0.041	0.273
D2 OT	VP	-0.380	-0.268	-0.157	5.64E-05
D1 OT	VP	-0.315	-0.203	-0.091	8.57E-04

Table S27. Post hoc comparisons of {S_K vs. P_K} linear classifier accuracy trained on PC1 across regions (Fig5L).

Table S28:

Source	Sum Sq.	d.f.	Mean Sq.	F	Prob>F
region	0.019	2	0.009	0.646	0.539
Error	0.206	14	0.015		
Total	0.225	16			

Table S28. One-way ANOVA for effect of region on {S_K vs. S_T} linear classifier accuracy trained on PC1-PC15 (Fig5L).

Table S29:

Group A	Group B	Lower Limit	A-B	Upper Limit	P-value
D2 OT	D1 OT	-0.203	-0.019	0.164	0.958
D2 OT	VP	-0.131	0.061	0.253	0.687
D1 OT	VP	-0.111	0.081	0.273	0.529

Table S29. Post hoc comparisons of {S_K vs. S_T} linear classifier accuracy trained on PC1-PC15 across regions (Fig5L).

Table S30:

Source	Sum Sq.	d.f.	Mean Sq.	F	Prob>F
spout	17.176	1	17.176	50.125	2.56E-07
S%	19.415	2	9.707	28.329	4.82E-07
spout:S%	11.533	2	5.767	16.829	2.71E-05
Error	8.224	24	0.343		
Total	56.348	29			

Table S30. 2-way ANOVA for effect of lick spout presence and sucrose contingency on anticipatory licking (Fig6C).

Table S31:

Group A	Group B	Lower Limit	A-B	Upper Limit	P-value
spout=0, S0.0	spout=1, S0.0	-1.205	-0.060	1.085	1
spout=0, S0.0	spout=0, S0.5	-1.335	-0.190	0.955	0.995
spout=0, S0.0	spout=1, S0.5	-2.725	-1.580	-0.435	3.24E-03
spout=0, S0.0	spout=0, S1.0	-1.595	-0.450	0.695	0.825
spout=0, S0.0	spout=1, S1.0	-4.685	-3.540	-2.395	1.66E-08
spout=1, S0.0	spout=0, S0.5	-1.275	-0.130	1.015	0.999
spout=1, S0.0	spout=1, S0.5	-2.665	-1.520	-0.375	4.80E-03
spout=1, S0.0	spout=0, S1.0	-1.535	-0.390	0.755	0.895
spout=1, S0.0	spout=1, S1.0	-4.625	-3.480	-2.335	2.29E-08
spout=0, S0.5	spout=1, S0.5	-2.535	-1.390	-0.245	1.11E-02
spout=0, S0.5	spout=0, S1.0	-1.405	-0.260	0.885	0.980
spout=0, S0.5	spout=1, S1.0	-4.495	-3.350	-2.205	4.70E-08
spout=1, S0.5	spout=0, S1.0	-0.015	1.130	2.275	5.44E-02
spout=1, S0.5	spout=1, S1.0	-3.105	-1.960	-0.815	2.58E-04
spout=0, S1.0	spout=1, S1.0	-4.235	-3.090	-1.945	2.07E-07

Table S31. Post hoc comparisons of anticipatory licking across spout presence and sucrose contingency (Fig6C).

Table S32:

**Source**	**Sum Sq.**	**d.f.**	**Mean Sq.**	**F**	**Prob>F**
days	466.659	5	93.332	1.199	0.308
valence	217.697	2	108.849	1.399	0.248
days:valence	671.044	10	67.104	0.862	0.569
Error	35948.767	462	77.811		
Total	37302.764	479			

Table S32. 2-way ANOVA for effect of imaging day and valence of odor on velocity during cue presentation (FigS6E).

Table S33:

**Source**	**Sum Sq.**	**d.f.**	**Mean Sq.**	**F**	**Prob>F**
days	851.615	5	170.323	0.660	0.654
valence	29846.379	2	14923.189	57.811	3.91E-23
days:valence	2979.836	10	297.984	1.154	0.320
Error	119259.047	462	258.136		
Total	153052.200	479			

Table S33. 2-way ANOVA for effect of imaging day and valence of odor on velocity during unconditioned stimulus (FigS6E).

Table S34:

Group A	Group B	Lower Limit	A-B	Upper Limit	P-value
d1,P	d1,X	5.990	20.970	35.951	1.51E-04
d1,P	d1,S	10.286	25.266	40.246	5.70E-07
d2,P	d2,X	-3.390	11.591	26.571	0.381
d2,P	d2,S	-2.310	12.670	27.651	0.225
d3,P	d3,X	-4.604	10.942	26.488	0.565
d3,P	d3,S	-1.079	14.466	30.012	0.104
d4,P	d4,X	-4.253	11.292	26.838	0.504
d4,P	d4,S	-1.729	13.817	29.363	0.155
d5,P	d5,X	-2.610	12.936	28.482	0.251
d5,P	d5,S	4.085	19.631	35.177	1.45E-03
d6,P	d6,X	-1.619	13.927	29.473	0.145
d6,P	d6,S	10.737	26.283	41.829	5.22E-07
d1,P	d2,P	-5.400	9.580	24.560	0.733
d1,P	d3,P	-5.063	10.203	25.469	0.660
d1,P	d4,P	-4.305	10.961	26.227	0.526
d1,P	d5,P	-6.954	8.311	23.577	0.913
d1,P	d6,P	-10.637	4.628	19.894	1
d2,P	d3,P	-14.643	0.623	15.889	1
d2,P	d4,P	-13.885	1.381	16.647	1
d2,P	d5,P	-16.534	-1.269	13.997	1
d2,P	d6,P	-20.217	-4.951	10.314	1
d3,P	d4,P	-14.788	0.758	16.304	1
d3,P	d5,P	-17.437	-1.892	13.654	1

d3,P	d6,P	-21.120	-5.574	9.971	0.999
d4,P	d5,P	-18.196	-2.650	12.896	1
d4,P	d6,P	-21.878	-6.333	9.213	0.995
d5,P	d6,P	-19.229	-3.683	11.863	1
d1,X	d2,X	-14.780	0.200	15.181	1
d1,X	d3,X	-15.091	0.175	15.441	1
d1,X	d4,X	-13.982	1.283	16.549	1
d1,X	d5,X	-14.989	0.277	15.543	1
d1,X	d6,X	-17.680	-2.414	12.851	1
d2,X	d3,X	-15.291	-0.025	15.240	1
d2,X	d4,X	-14.183	1.083	16.349	1
d2,X	d5,X	-15.189	0.077	15.342	1
d2,X	d6,X	-17.881	-2.615	12.651	1
d3,X	d4,X	-14.437	1.108	16.654	1
d3,X	d5,X	-15.444	0.102	15.648	1
d3,X	d6,X	-18.135	-2.589	12.956	1
d4,X	d5,X	-16.552	-1.006	14.540	1
d4,X	d6,X	-19.244	-3.698	11.848	1
d5,X	d6,X	-18.237	-2.691	12.854	1
d1,S	d2,S	-17.996	-3.015	11.965	1
d1,S	d3,S	-15.862	-0.597	14.669	1
d1,S	d4,S	-15.753	-0.488	14.778	1
d1,S	d5,S	-12.589	2.677	17.942	1
d1,S	d6,S	-9.620	5.646	20.911	0.998
d2,S	d3,S	-12.847	2.419	17.685	1

d2,S	d4,S	-12.738	2.528	17.794	1
d2,S	d5,S	-9.574	5.692	20.958	0.998
d2,S	d6,S	-6.605	8.661	23.927	0.880
d3,S	d4,S	-15.437	0.109	15.655	1
d3,S	d5,S	-12.273	3.273	18.819	1
d3,S	d6,S	-9.304	6.242	21.788	0.996
d4,S	d5,S	-12.382	3.164	18.710	1
d4,S	d6,S	-9.413	6.133	21.679	0.997
d5,S	d6,S	-12.577	2.969	18.515	1

Table S34. Post hoc comparisons of velocity during unconditioned stimulus across imaging days and valence of odor (FigS6E).

Table S35:

Source	Sum Sq.	d.f.	Mean Sq.	F	Prob>F
days	0.293	5	0.059	12.301	8.84E-11
valence	0.014	2	0.007	1.523	0.220
days:valence	0.121	10	0.012	2.546	5.95E-03
Error	1.313	276	0.005		
Total	1.739	293			

Table S35. 2-way ANOVA for effect of imaging day and valence of odor on relative eye size during cue presentation (FigS6G).

Table S36:

Source	Sum Sq.	d.f.	Mean Sq.	F	Prob>F
days	0.178	5	0.036	4.529	5.51E-04
valence	0.040	2	0.020	2.574	0.078
days:valence	0.167	10	0.017	2.123	2.29E-02
Error	2.167	276	0.008		
Total	2.547	293			

Table S36. 2-way ANOVA for effect of imaging day and valence of odor on relative eye size during unconditioned stimulus (FigS6G).

Table S37:

Source	Sum Sq.	d.f.	Mean Sq.	F	Prob>F
ket	0.051	1	0.051	3.828	0.051
val.	0.787	2	0.393	29.766	2.48E-12
reg.	6.62E-03	2	0.003	0.250	0.779
day	0.928	2	0.464	35.101	3.57E-14
ket:val.	0.024	2	0.012	0.911	0.403
ket:reg.	0.018	2	0.009	0.671	0.512
ket:day	0.057	2	0.029	2.163	0.117
val.:reg.	0.622	4	0.155	11.763	8.94E-09
val.:day	0.264	4	0.066	4.995	6.85E-04
reg.:day	0.328	4	0.082	6.212	8.79E-05
ket:val.:reg.	0.054	4	0.014	1.025	0.395
ket:val.:day	0.095	4	0.024	1.796	0.130
ket:reg.:day	0.014	4	0.004	0.272	0.896
val.:reg.:day	0.241	8	0.030	2.277	0.023
ket:val.:reg.:day	0.062	8	0.008	0.582	0.792
Error	3.331	252	0.013		
Total	6.670	305			

Table S37. 4-way ANOVA for effect of imaging day, valence, functional group, and region on the percentage of neurons responsive to a given odor (FigS8A).

Table S38:

**Formula:**					
Fmag ~ 1 + reg*day + reg*val + day*val + reg:day:val + (1 | id)					
**Model information**					
**# of observations:**	**Fixed effects coefficients:**	**Random effect coefficients:**		**Covariance parameters:**	
11,160	12	17		2	
**Model fit statistics:**					
**AIC**	**BIC**	**Log Likelihood**		**Deviance**	
9050.1	9152.6	-4511.1		9022.1	
**Fixed effects coefficients (95% CIs):**					
**Name**	**Estimate**	**SE**	**tStat**	**DF**	**pValue**
intercept	0.469	0.0155	30.228	11,148	5.43E-193
reg_D1	-0.0222	0.0205	-1.0829	11,148	0.279
reg_VP	0.0192	0.0255	0.753	11,148	0.452
day	-0.0140	0.00707	-1.973	11,148	0.0485
val	-0.0244	0.0190	-1.286	11,148	0.198
reg_D1:day	0.00806	0.00947	0.852	11,148	0.394
reg_VP:day	-0.0101	0.0117	-0.862	11,148	0.389
reg_D1:val	-0.00659	0.0251	-0.262	11,148	0.793
reg_VP:val	-0.0359	0.0312	-1.150	11,148	0.250
day:val	0.00799	0.00866	0.922	11,148	0.356
reg_D1:day:val	0.0294	0.0116	2.539	11,148	0.0111
reg_VP:day:val	0.0826	0.0143	5.762	11,148	8.5E-9

Table S38. Linear model of the fixed effects of region, imaging day, and valence and the random effect of individual animal on |ΔΔF/F| (FigS9A).

Table S39:

**Formula:**					
auROC {S vs. X|P} ~ 1 + region*day + (1 | id)					
**Model information**					
**Number of observations:**	**Fixed effects coefficients:**	**Random effect coefficients:**		**Covariance parameters:**	
1860	6	17		2	
**Model fit statistics:**					
**AIC**	**BIC**	**Log Likelihood**		**Deviance**	
-4303.7	-4259.5	2159.8		-4319.7	
**Fixed effects coefficients (95% CIs):**					
**Name**	**Estimate**	**SE**	**tStat**	**DF**	**pValue**
intercept	0.617	0.009	68.007	1854	0
reg_D1	-0.002	0.012	-0.186	1854	0.853
reg_VP	-0.037	0.014	-2.665	1854	7.76E-03
day	0.003	0.001	1.874	1854	0.061
reg_D1:day	0.007	0.002	3.484	1854	5.06E-04
reg_VP:day	0.029	0.002	12.056	1854	2.80E-32

Table S39. Linear model of the fixed effects of region and imaging day, and the random effect of individual animals on the auROC of single-neuron {S vs. X|P} classifiers (FigS10F).

Table S40:

Source	Sum Sq.	d.f.	Mean Sq.	F	Prob>F
region	0.030	2	0.015	14.686	1.46E-05
day	0.050	2	0.025	24.336	9.58E-08
region:day	0.041	4	0.010	10.001	8.88E-06
Error	0.043	42	0.001		
Total	0.158	50			

Table S40. 2-way ANOVA for effect of imaging day and region on the median auROC value of {S vs. X|P} classifiers for each animal (FigS10F).

Table S41:

Group A	Group B	Lower Limit	A-B	Upper Limit	P-value
D2 OT,d1	D2 OT,d3	-0.072	-0.012	0.049	0.999
D2 OT,d1	D2 OT,d6	-0.075	-0.014	0.046	0.997
D2 OT,d3	D2 OT,d6	-0.063	-0.003	0.058	1
D1 OT,d3	D1 OT,d6	-0.072	-0.012	0.049	0.999
D1 OT,d1	D1 OT,d3	-0.091	-0.030	0.030	0.779
D1 OT,d1	D1 OT,d6	-0.102	-0.042	0.019	0.387
VP,d1	VP,d3	-0.130	-0.064	0.002	6.62E-02
VP,d1	VP,d6	-0.241	-0.174	-0.108	2.83E-09
VP,d3	VP,d6	-0.177	-0.111	-0.044	7.91E-05
D2 OT,d1	D1 OT,d1	-0.056	0.005	0.065	1
D2 OT,d1	VP,d1	-0.050	0.013	0.076	0.999
D1 OT,d1	VP,d1	-0.055	0.008	0.072	1
D2 OT,d3	D1 OT,d3	-0.074	-0.014	0.047	0.998
D2 OT,d3	VP,d3	-0.103	-0.039	0.024	0.537
D1 OT,d3	VP,d3	-0.089	-0.026	0.038	0.921
D2 OT,d6	D1 OT,d6	-0.083	-0.023	0.038	0.948
D2 OT,d6	VP,d6	-0.210	-0.147	-0.084	7.68E-08
D1 OT,d6	VP,d6	-0.188	-0.124	-0.061	3.44E-06

Table S41. Post hoc comparison of the median auROC value for {S vs. X|P} across imaging day and region (FigS10F).

Table S42:

**Formula:**					
auROC {S_K vs. S_T} ~ 1 + region*day + (1 | id)					
**Model information**					
**Number of observations:**	**Fixed effects coefficients:**	**Random effect coefficients:**		**Covariance parameters:**	
1860	6	17		2	
**Model fit statistics:**					
**AIC**	**BIC**	**Log Likelihood**		**Deviance**	
-2908.8	-2864.5	1462.4		-2924.8	
**Fixed effects coefficients (95% CIs):**					
**Name**	**Estimate**	**SE**	**tStat**	**DF**	**pValue**
intercept	0.636	0.015	42.040	1854	7.72E-272
region_D1	-0.020	0.021	-0.962	1854	0.336
region_VP	-0.024	0.024	-1.014	1854	0.311
day	9.67E-04	5.25E-03	0.184	1854	0.854
region_D1:day	0.011	0.007	1.622	1854	0.105
region_VP:day	-0.003	0.009	-0.346	1854	0.730

Table S42. Linear model of the fixed effects of region and imaging day, and the random effect of individual animals on the auROC of single-neuron {S_K vs. S_T} classifiers (FigS10G).

Table S43:

Source	Sum Sq.	d.f.	Mean Sq.	F	Prob>F
region	0.011	2	5.54E-03	2.793	0.073
day	2.91E-04	2	1.46E-04	0.073	0.929
region:day	3.66E-03	4	9.16E-04	0.462	0.763
Error	0.083	42	1.98E-03		
Total	0.098	50			

Table S43. 2-way ANOVA for effect of imaging day and region on the median auROC value of {S_K vs. S_T} classifiers for each animal (FigS10G).

Table S44:

Formula:					
score ~ 1 + region*day + (1 | id)					
**Model information**					
**Number of observations:**	**Fixed effects coefficients:**	**Random effect coefficients:**		**Covariance parameters:**	
1860	6	17		2	
**Model fit statistics:**					
**AIC**	**BIC**	**Log Likelihood**		**Deviance**	
-3219.9	-3175.7	1618		-3235.9	
**Fixed effects coefficients (95% CIs):**					
**Name**	**Estimate**	**SE**	**tStat**	**DF**	**pValue**
intercept	-0.023	0.015	-1.563	1854	0.118
region_D1	4.19E-03	0.020	0.204	1854	0.838
region_VP	-0.062	0.023	-2.648	1854	8.16E-03
day	5.84E-03	4.83E-03	1.209	1854	0.227
region_D1:day	6.40E-03	6.45E-03	0.991	1854	0.322
region_VP:day	0.075	7.98E-03	9.384	1854	1.80E-20

Table S44: Linear model of the fixed effects of region and imaging day, and the random effect of individual animals on the single-neuron valence scores (FigS10H).

Table S45:

Source	Sum Sq.	d.f.	Mean Sq.	F	Prob>F
reg	0.070	2	0.035	13.163	3.65E-05
day	0.032	2	0.016	6.073	4.82E-03
reg:day	0.045	4	0.011	4.264	5.50E-03
Error	0.111	42	2.65E-03		
Total	0.253	50			

Table S45. 2-way ANOVA for effect of imaging day and region on the median valence score for each animal (FigS10H).

Table S46:

Group A	Group B	Lower Limit	A-B	Upper Limit	P-value
D2 OT,d1	D2 OT,d3	-0.100	-0.003	0.094	1
D2 OT,d1	D2 OT,d6	-0.109	-0.012	0.086	1
D2 OT,d3	D2 OT,d6	-0.106	-0.008	0.089	1
D1 OT,d1	D1 OT,d3	-0.100	-0.002	0.095	1
D1 OT,d1	D1 OT,d6	-0.102	-0.005	0.092	1
D1 OT,d3	D1 OT,d6	-0.100	-0.003	0.095	1
VP,d1	VP,d3	-0.157	-0.051	0.056	0.819
VP,d1	VP,d6	-0.271	-0.165	-0.058	2.87E-04
VP,d3	VP,d6	-0.220	-0.114	-0.007	2.87E-02
D2 OT,d1	D1 OT,d1	-0.112	-0.015	0.082	1
D2 OT,d1	VP,d1	-0.122	-0.020	0.082	1
D1 OT,d1	VP,d1	-0.107	-0.005	0.097	1
D2 OT,d3	D1 OT,d3	-0.111	-0.014	0.083	1
D2 OT,d3	VP,d3	-0.169	-0.067	0.034	0.447
D1 OT,d3	VP,d3	-0.155	-0.053	0.049	0.736
D2 OT,d6	D1 OT,d6	-0.105	-0.008	0.089	1
D2 OT,d6	VP,d6	-0.275	-0.173	-0.071	6.07E-05
D1 OT,d6	VP,d6	-0.266	-0.164	-0.062	1.41E-04

Table S46. Post hoc comparison of the median valence scores across imaging day and region (FigS10H).

Table S47:

**Source**	**Sum Sq.**	**d.f.**	**Mean Sq.**	**F**	**Prob>F**
reg	0.003	2	0.002	4.286	2.02E-02
day	0.006	2	0.003	7.181	2.08E-03
reg:day	0.004	4	0.001	2.622	4.82E-02
Error	0.017	42	0.000		
Total	0.030	50			

Table S47. 2-way ANOVA for effect of imaging day and region on the median single-neuron MNR accuracy for each animal (FigS11B).

Table S48:

Group A	Group B	Lower Limit	A-B	Upper Limit	P-value
D2 OT,d1	D2 OT,d3	-0.051	-0.013	0.024	0.960
D2 OT,d1	D2 OT,d6	-0.045	-0.007	0.031	0.999
D2 OT,d3	D2 OT,d6	-0.032	0.006	0.044	1
D1 OT,d1	D1 OT,d3	-0.047	-0.010	0.028	0.996
D1 OT,d1	D1 OT,d6	-0.050	-0.012	0.026	0.981
D1 OT,d3	D1 OT,d6	-0.040	-0.002	0.036	1
VP,d1	VP,d3	-0.073	-0.031	0.011	0.294
VP,d1	VP,d6	-0.099	-0.058	-0.016	1.47E-03
VP,d3	VP,d6	-0.068	-0.027	0.015	0.490
D2 OT,d1	D1 OT,d1	-0.052	-0.014	0.024	0.953
D2 OT,d1	VP,d1	-0.037	0.003	0.043	1
D1 OT,d1	VP,d1	-0.023	0.017	0.057	0.896
D2 OT,d3	D1 OT,d3	-0.048	-0.010	0.028	0.994
D2 OT,d3	VP,d3	-0.054	-0.014	0.025	0.955
D1 OT,d3	VP,d3	-0.044	-0.005	0.035	1
D2 OT,d6	D1 OT,d6	-0.057	-0.019	0.019	0.797
D2 OT,d6	VP,d6	-0.087	-0.047	-0.008	9.47E-03
D1 OT,d6	VP,d6	-0.069	-0.029	0.011	0.329

Table S48. Post hoc comparison of median single-neuron MNR accuracy across imaging day and region (FigS11B).

Table S49:

Source	Sum Sq.	d.f.	Mean Sq.	F	Prob>F
reg	2.04E-08	2	1.02E-08	0.040	0.961
day	7.71E-07	2	3.86E-07	1.519	0.231
reg:day	1.22E-06	4	3.05E-07	1.200	0.325
Error	1.07E-05	42	2.54E-07		
Total	1.26E-05	50			

Table S49. 2-way ANOVA for effect of imaging day and region on the median single-neuron MNR shuffled accuracy for each animal (FigS11C).

Table S50:

Source	Sum Sq.	d.f.	Mean Sq.	F	Prob>F
reg	0.074	2	0.037	27.720	2.11E-08
day	0.019	2	9.43E-03	7.066	2.26E-03
reg:day	0.011	4	2.65E-03	1.986	1.14E-01
Error	0.056	42	1.33E-03		
Total	0.157	50			

Table S50. 2-way ANOVA for effect of imaging day and region on the median S-cue/S-cue confusion for each animal (FigS11D).

Table S51:

Group A	Group B	Lower Limit	A-B	Upper Limit	P-value
D2 OT,d1	D1 OT,d1	-0.090	-0.021	0.048	0.985
D2 OT,d1	VP,d1	-0.132	-0.060	0.012	0.174
D1 OT,d1	VP,d1	-0.111	-0.039	0.033	0.700
D2 OT,d3	D1 OT,d3	-0.095	-0.026	0.043	0.940
D2 OT,d3	VP,d3	-0.156	-0.084	-0.011	1.30E-02
D1 OT,d3	VP,d3	-0.130	-0.057	0.015	0.223
D2 OT,d6	D1 OT,d6	-0.084	-0.015	0.054	0.998
D2 OT,d6	VP,d6	-0.204	-0.132	-0.059	1.55E-05
D1 OT,d6	VP,d6	-0.189	-0.116	-0.044	1.46E-04
D2 OT,d1	D2 OT,d3	-0.079	-0.010	0.059	1
D2 OT,d1	D2 OT,d6	-0.094	-0.025	0.044	0.955
D2 OT,d3	D2 OT,d6	-0.084	-0.015	0.054	0.998
D1 OT,d1	D1 OT,d3	-0.084	-0.015	0.054	0.998
D1 OT,d1	D1 OT,d6	-0.088	-0.019	0.049	0.990
D1 OT,d3	D1 OT,d6	-0.073	-0.004	0.065	1
VP,d1	VP,d3	-0.109	-0.033	0.042	0.874
VP,d1	VP,d6	-0.172	-0.097	-0.021	4.11E-03
VP,d3	VP,d6	-0.139	-0.063	0.012	0.165

Table S51. Post hoc comparison of median S-cue/S-cue confusion across imaging day and region (FigS11D).

Table S52:

**Source**	**Sum Sq.**	**d.f.**	**Mean Sq.**	**F**	**Prob>F**
reg	1.60E-03	2	7.99E-04	0.681	0.512
day	0.017	2	8.71E-03	7.426	1.73E-03
reg:day	2.53E-03	4	6.31E-04	0.538	0.708
Error	0.049	42	1.17E-03		
Total	0.070	50			

Table S52. 2-way ANOVA for effect of imaging day and region on the median confusion within functional groups for each animal (FigS11E).

Table S53:

Group A	Group B	Lower Limit	A-B	Upper Limit	P-value
D2 OT,d1	D2 OT,d3	-0.055	0.009	0.074	1.000
D2 OT,d1	D2 OT,d6	-0.033	0.031	0.096	0.804
D2 OT,d3	D2 OT,d6	-0.042	0.022	0.087	0.967
D1 OT,d1	D1 OT,d3	-0.063	0.002	0.066	1
D1 OT,d1	D1 OT,d6	-0.034	0.031	0.095	0.828
D1 OT,d3	D1 OT,d6	-0.036	0.029	0.093	0.871
VP,d1	VP,d3	-0.060	0.011	0.082	1
VP,d1	VP,d6	-0.005	0.066	0.136	0.089
VP,d3	VP,d6	-0.016	0.054	0.125	0.255
D2 OT,d1	D1 OT,d1	-0.061	0.004	0.068	1
D2 OT,d1	VP,d1	-0.067	0.001	0.069	1
D1 OT,d1	VP,d1	-0.071	-0.003	0.065	1
D2 OT,d3	D1 OT,d3	-0.068	-0.004	0.061	1
D2 OT,d3	VP,d3	-0.065	0.003	0.070	1
D1 OT,d3	VP,d3	-0.061	0.006	0.074	1
D2 OT,d6	D1 OT,d6	-0.062	0.003	0.067	1
D2 OT,d6	VP,d6	-0.033	0.035	0.103	0.756
D1 OT,d6	VP,d6	-0.036	0.032	0.100	0.828

Table S53. Post hoc comparison of median within-function group confusion across imaging day and region (FigS11E).

Table S54:

Source	Sum Sq.	d.f.	Mean Sq.	F	Prob>F
region	0.010	2	4.92E-03	0.868	0.427
day	0.178	2	0.089	15.734	7.95E-06
region:day	0.064	4	0.016	2.846	3.56E-02
Error	0.238	42	5.67E-03		
Total	0.476	50			

Table S54. 2-way ANOVA for effect of imaging day and region on the mean accuracy for linear classification of {S vs. X} using population data (FigS12B).

Table S55:

Group A	Group B	Lower Limit	A-B	Upper Limit	P-value
D1 OT,d1	D2 OT,d1	-0.108	0.034	0.176	0.997
D1 OT,d1	VP,d1	-0.018	0.131	0.280	0.127
D2 OT,d1	VP,d1	-0.052	0.097	0.246	0.476
D1 OT,d3	D2 OT,d3	-0.081	0.061	0.203	0.889
D1 OT,d3	VP,d3	-0.153	-0.004	0.145	1
D2 OT,d3	VP,d3	-0.214	-0.065	0.084	0.880
D1 OT,d6	D2 OT,d6	-0.142	0.000	0.142	1
D1 OT,d6	VP,d6	-0.202	-0.053	0.096	0.960
D2 OT,d6	VP,d6	-0.202	-0.053	0.096	0.960
D1 OT,d1	D1 OT,d3	-0.190	-0.048	0.094	0.971
D1 OT,d1	D1 OT,d6	-0.214	-0.072	0.070	0.765
D1 OT,d3	D1 OT,d6	-0.166	-0.024	0.118	1
D2 OT,d1	D2 OT,d3	-0.163	-0.021	0.121	1
D2 OT,d1	D2 OT,d6	-0.248	-0.106	0.036	0.288
D2 OT,d3	D2 OT,d6	-0.227	-0.085	0.057	0.575
VP,d1	VP,d3	-0.338	-0.183	-0.027	1.12E-02
VP,d1	VP,d6	-0.411	-0.256	-0.100	1.02E-04
VP,d3	VP,d6	-0.229	-0.073	0.082	0.830

Table S55. Post hoc comparison of mean {S vs. X} accuracy across imaging day and region (FigS12B).

Table S56:

Source	Sum Sq.	d.f.	Mean Sq.	F	Prob>F
region	0.020	2	0.010	1.776	0.182
day	0.255	2	0.128	22.389	2.41E-07
region:day	0.042	4	0.010	1.822	0.143
Error	0.240	42	5.71E-03		
Total	0.543	50			

Table S56. 2-way ANOVA for effect of imaging day and region on the mean accuracy for linear classification of {S vs. P} using population data (FigS12C).

Table S57:

Group A	Group B	Lower Limit	A-B	Upper Limit	P-value
D1 OT,d1	D2 OT,d1	-0.104	0.038	0.181	0.993
D1 OT,d1	VP,d1	-0.051	0.098	0.248	0.453
D2 OT,d1	VP,d1	-0.089	0.060	0.210	0.920
D1 OT,d3	D2 OT,d3	-0.057	0.085	0.228	0.579
D1 OT,d3	VP,d3	-0.134	0.015	0.165	1
D2 OT,d3	VP,d3	-0.219	-0.070	0.079	0.835
D1 OT,d6	D2 OT,d6	-0.124	0.019	0.161	1
D1 OT,d6	VP,d6	-0.192	-0.043	0.107	0.989
D2 OT,d6	VP,d6	-0.211	-0.062	0.088	0.910
D1 OT,d1	D1 OT,d3	-0.233	-0.090	0.052	0.506
D1 OT,d1	D1 OT,d6	-0.262	-0.119	0.023	0.165
D1 OT,d3	D1 OT,d6	-0.172	-0.029	0.113	0.999
D2 OT,d1	D2 OT,d3	-0.186	-0.043	0.099	0.985
D2 OT,d1	D2 OT,d6	-0.281	-0.139	0.004	0.061
D2 OT,d3	D2 OT,d6	-0.238	-0.096	0.047	0.426
VP,d1	VP,d3	-0.329	-0.173	-0.017	1.97E-02
VP,d1	VP,d6	-0.417	-0.261	-0.105	7.71E-05
VP,d3	VP,d6	-0.244	-0.088	0.069	0.662

Table S57. Post hoc comparison of mean {S vs. P} accuracy across imaging day and region (FigS12C).

Table S58:

Source	Sum Sq.	d.f.	Mean Sq.	F	Prob>F
region	0.085	2	0.042	3.600	0.036
day	0.033	2	0.017	1.415	0.254
region:day	0.042	4	0.010	0.890	0.478
Error	0.493	42	0.012		
Total	0.655	50			

Table S58. 2-way ANOVA for effect of imaging day and region on the accuracy for linear classification of {S_K vs. S_T} using population data (FigS12D).

Table S59:

Group A	Group B	Lower Limit	A-B	Upper Limit	P-value
D1 OT,d1	D2 OT,d1	-0.202	0.003	0.207	1
D1 OT,d1	VP,d1	-0.121	0.094	0.308	0.879
D2 OT,d1	VP,d1	-0.123	0.091	0.306	0.896
D1 OT,d3	D2 OT,d3	-0.238	-0.033	0.171	1
D1 OT,d3	VP,d3	-0.216	-0.002	0.213	1
D2 OT,d3	VP,d3	-0.183	0.032	0.246	1
D1 OT,d6	D2 OT,d6	-0.307	-0.103	0.102	0.776
D1 OT,d6	VP,d6	-0.135	0.079	0.294	0.950
D2 OT,d6	VP,d6	-0.032	0.182	0.397	0.153
D1 OT,d1	D1 OT,d3	-0.199	0.006	0.210	1
D1 OT,d1	D1 OT,d6	-0.227	-0.022	0.182	1
D1 OT,d3	D1 OT,d6	-0.232	-0.028	0.177	1
D2 OT,d1	D2 OT,d3	-0.235	-0.031	0.174	1
D2 OT,d1	D2 OT,d6	-0.332	-0.128	0.077	0.525
D2 OT,d3	D2 OT,d6	-0.302	-0.097	0.107	0.823
VP,d1	VP,d3	-0.314	-0.090	0.134	0.922
VP,d1	VP,d6	-0.261	-0.037	0.187	1
VP,d3	VP,d6	-0.171	0.053	0.277	0.997

Table S59. Post hoc comparison of {S_K vs. S_T} accuracy across imaging day and region (FigS12D).
