# Supplementary material for: Transformation of value signaling in a striatopallidal circuit
Source: bioRxiv. 2023 Aug 3:2023.08.01.551547. Preprint. [Version 1] doi: 10.1101/2023.08.01.551547 (PMC10418236; doi:10.1101/2023.08.01.551547)
Supplement: Supplementary file 1 [file NIHPP2023.08.01.551547v1-supplement-1.pdf]

bioRxiv preprint doi: https://doi.org/10.1101/2023.08.01.551547; this version posted August 3, 2023. The copyright holder for this preprint (which was not certified by peer review) is the author/funder, who has granted bioRxiv a license to display the preprint in perpetuity. It is made available under aCC-BY 4.0 International license.

Figure S1:

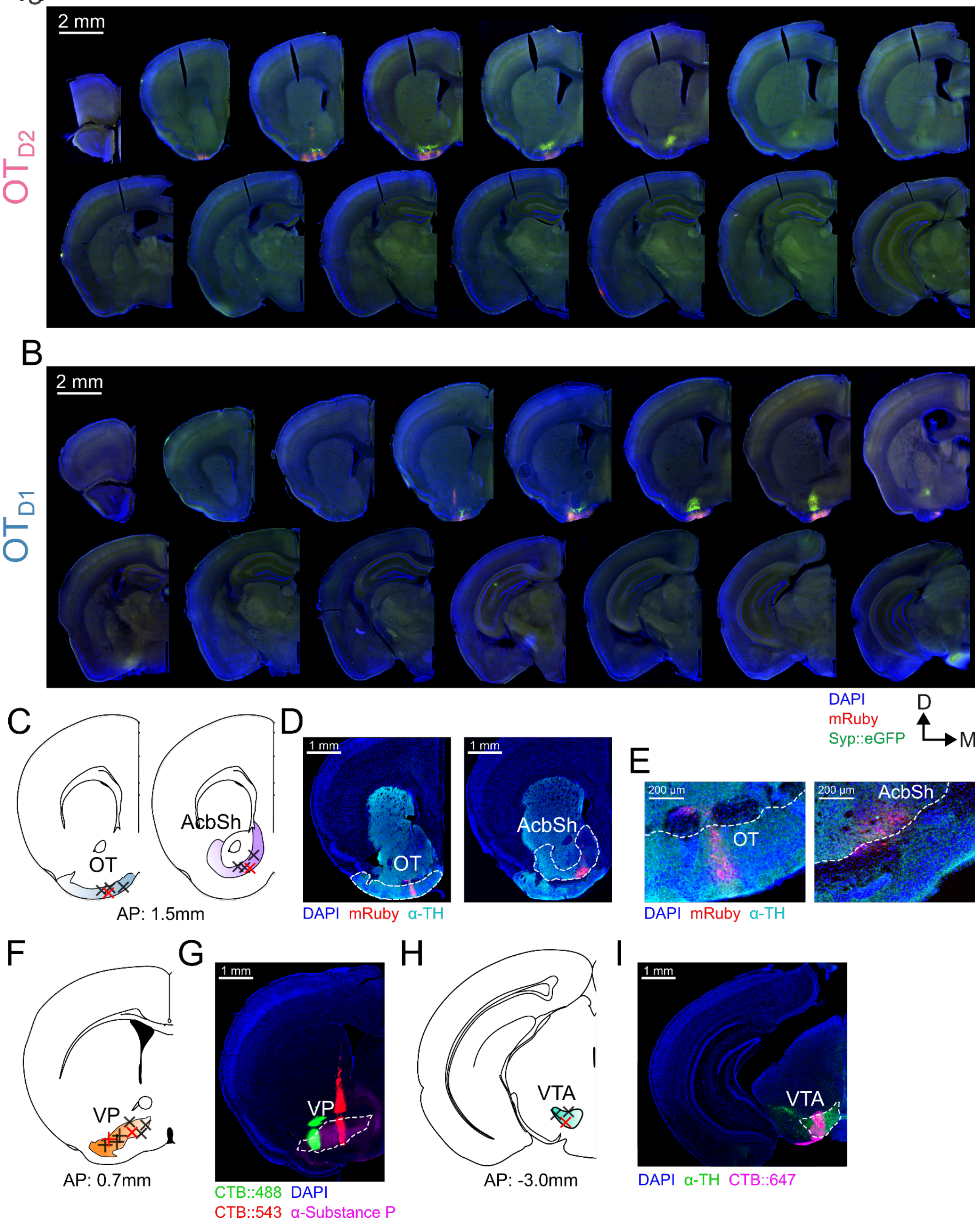

bioRxiv preprint doi: https://doi.org/10.1101/2023.08.01.551547; this version posted August 3, 2023. The copyright holder for this preprint (which was not certified by peer review) is the author/funder, who has granted bioRxiv a license to display the preprint in perpetuity. It is made available under aCC-BY 4.0 International license.

Figure S1. OT_{D1} and OT_{D2} primarily project to the lateral portion of the VP.
(**A**) Serial coronal sections from a representative experiment where AAVDJ-hSyn-FLEX-mRuby-T2A-syn-eGFP virus was injected into the anterior OT of an *Adora2a*-Cre mouse. Sections are roughly 400μm apart from each other and range from +2.5mm to -3.0mm relative to bregma. (**B**) Same as (A) but in a *Drd1*-Cre mouse. (**C**) Schematic showing the centroids of 4 injection sites for OT and AcbSh anterograde tracing experiments. (**D**, **E**) Representative images of the injection sites shown in (C). Sections were counterstained with α-TH to delineate the boundary between the striatum and the rostral ventral pallidum. The centroids of these samples are marked as red x's in (C). (**F**) Schematic showing the centroids of 4 CTB injection sites for lateral and medial VP. CTB::488 injection to the lateral VP are marked by +'s whereas CTB::543 injection to the medial VP are marked by x's. (**G**) Representative image of the injection sites shown in (F). Sections were counterstained with α-Substance P to mark the boundary of the VP. The centroids of this sample are marked by the red + and red x in (F). (**H**) Schematic showing the centroids of 3 CTB injection sites for VTA. (**I**) Representative image of the injection sites shown in (H). Sections were counterstained with α-TH to mark the boundary of the VTA.

bioRxiv preprint doi: https://doi.org/10.1101/2023.08.01.551547; this version posted August 3, 2023. The copyright holder for this preprint (which was not certified by peer review) is the author/funder, who has granted bioRxiv a license to display the preprint in perpetuity. It is made available under aCC-BY 4.0 International license.

Figure S2:

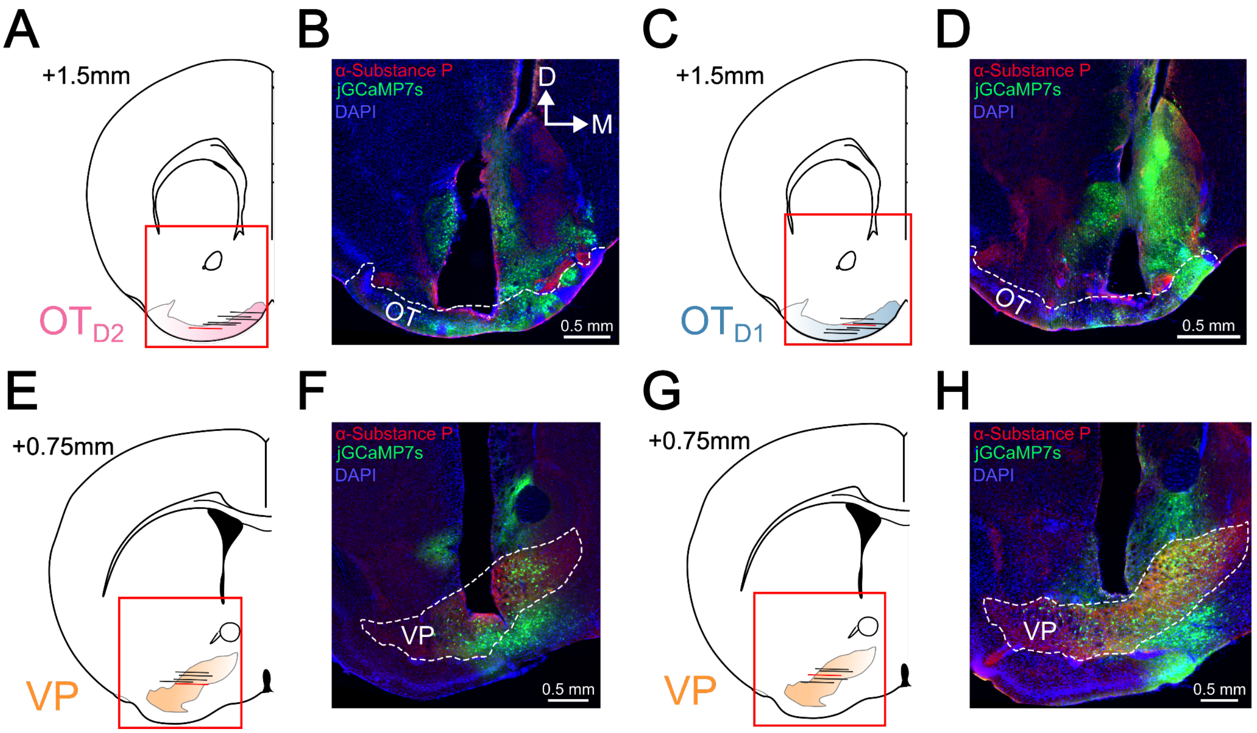

bioRxiv preprint doi: https://doi.org/10.1101/2023.08.01.551547; this version posted August 3, 2023. The copyright holder for this preprint (which was not certified by peer review) is the author/funder, who has granted bioRxiv a license to display the preprint in perpetuity. It is made available under aCC-BY 4.0 International license.

Figure S2 Histological verification of lens implant location.
(**A**) Schematic showing the center, along the AP axis, of the implanted GRIN lens in 6 OT_{D2} jGCaMP7s animals. (**B**) Representative image of lens implant sites shown in (A). Sections were counterstained with α-Substance P to delineate the boundary of the VP. This sample is marked by a red horizontal line in (A). (**C**) Schematic as in (A) for 6 OT_{D1} jGCaMP7s animals. (**D**) Representative image of lens implant sites shown in (C). (**E**) Schematic as in (A) for 5 VP jGCaMP7s animals. (**F**) Representative image of lens implant sites shown in (E). (**G**) Schematic as in (A) for 5 VP jGCaMP7s animals recorded during the lick spout retraction paradigm (Figure 6). (**H**) Representative image of lens implant sites shown in (G). Sections were counterstained with α-Substance P to delineate the boundary of the VP. This sample is marked by a red horizontal line in (G).

bioRxiv preprint doi: https://doi.org/10.1101/2023.08.01.551547; this version posted August 3, 2023. The copyright holder for this preprint (which was not certified by peer review) is the author/funder, who has granted bioRxiv a license to display the preprint in perpetuity. It is made available under aCC-BY 4.0 International license.

Figure S3:

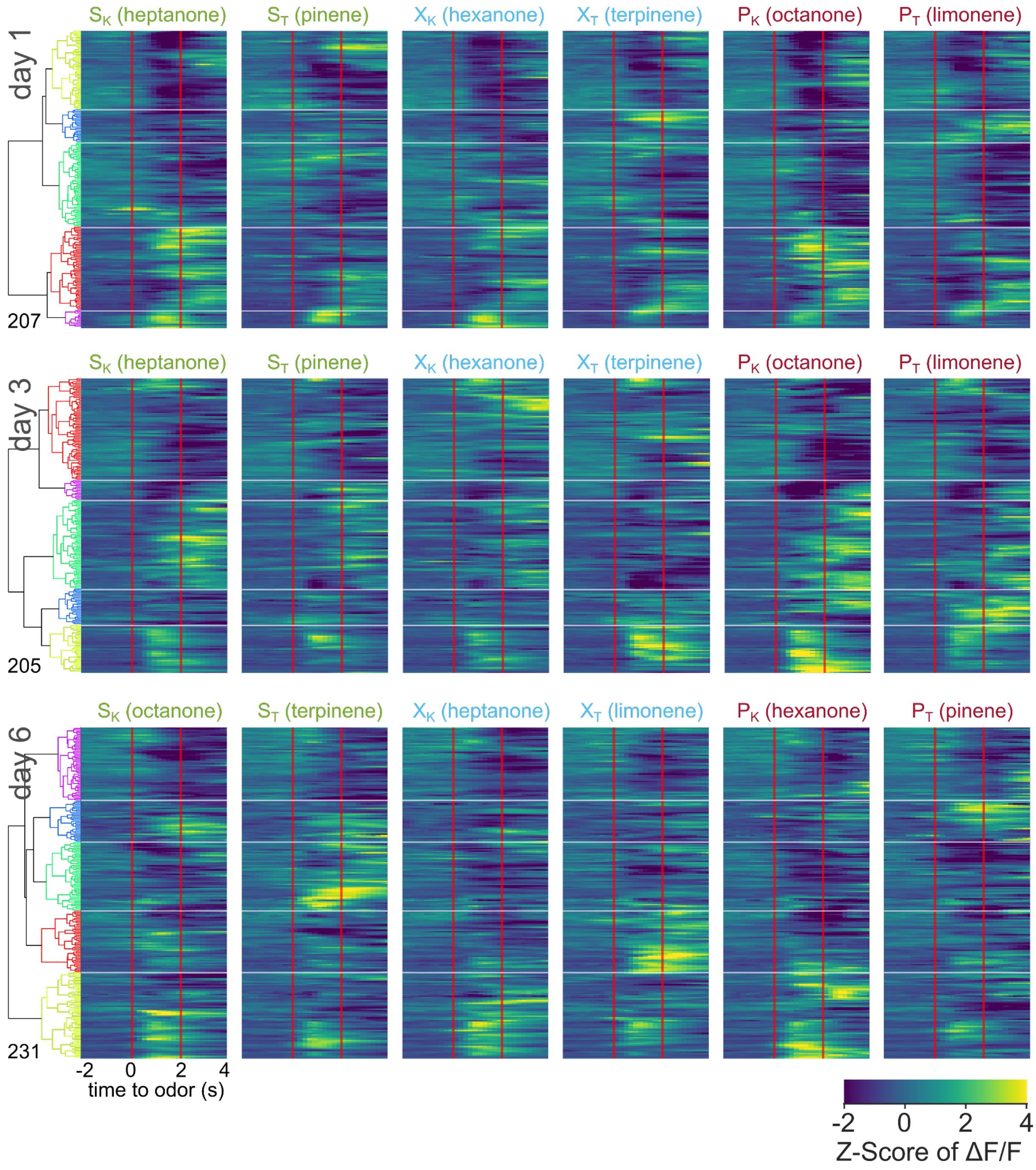

bioRxiv preprint doi: https://doi.org/10.1101/2023.08.01.551547; this version posted August 3, 2023. The copyright holder for this preprint (which was not certified by peer review) is the author/funder, who has granted bioRxiv a license to display the preprint in perpetuity. It is made available under aCC-BY 4.0 International license.

Figure S3. Pooled averaged-over-trials neural activity of all neurons from OT_{D2} animals across days. Heatmap of odor-evoked activity in OT_{D2} neurons from day 1, day 3, and day 6 of imaging. The fluorescence measurements from each neuron were averaged over trials, Z-scored, then pooled for hierarchical clustering. Neurons are grouped by similarity, with the dendrogram shown on the right. Horizontal white lines demarcate the boundaries between the 6 clusters. Odor delivered at 0-2 seconds marked by vertical red lines. From left to right, the columns represent neural responses to sucrose-paired ketone and terpene, control ketone and terpene, and airpuff-paired ketone and terpene (S_K, S_T, X_K, X_T, P_K, P_T). Data is pooled from 6 animals.

bioRxiv preprint doi: https://doi.org/10.1101/2023.08.01.551547; this version posted August 3, 2023. The copyright holder for this preprint (which was not certified by peer review) is the author/funder, who has granted bioRxiv a license to display the preprint in perpetuity. It is made available under aCC-BY 4.0 International license.

Figure S4:

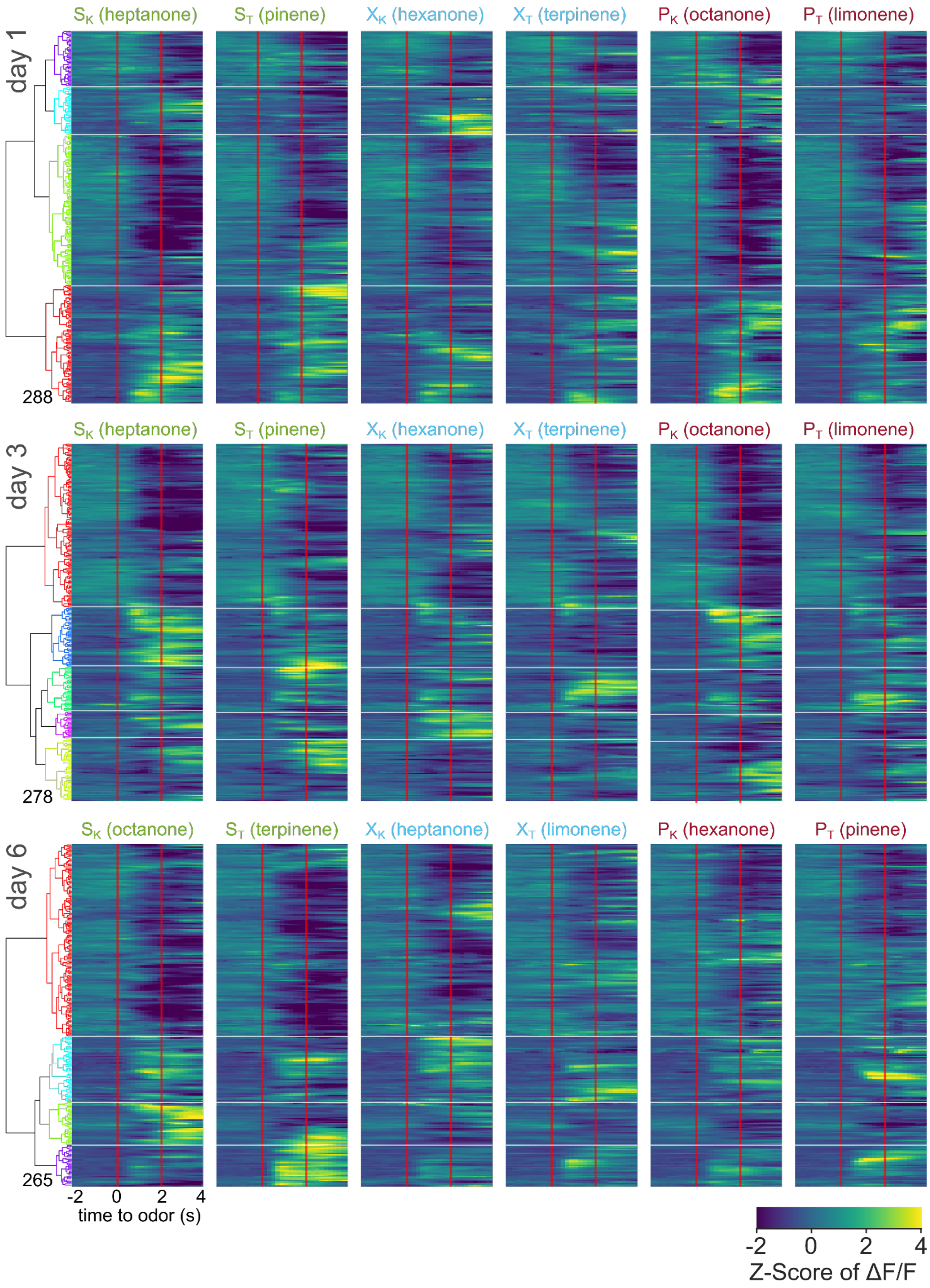

bioRxiv preprint doi: https://doi.org/10.1101/2023.08.01.551547; this version posted August 3, 2023. The copyright holder for this preprint (which was not certified by peer review) is the author/funder, who has granted bioRxiv a license to display the preprint in perpetuity. It is made available under aCC-BY 4.0 International license.

Figure S4. Pooled averaged-over-trials neural activity of all neurons from OT_{D1} animals across days. Heatmap of odor-evoked activity in OT_{D1} neurons from day 1, day 3, and day 6 of imaging.. The fluorescence measurements from each neuron were averaged over trials, Z-scored, then pooled for hierarchical clustering. Neurons are grouped by similarity, with the dendrogram shown on the right. Horizontal white lines demarcate the boundaries between the 6 clusters. Odor delivered at 0-2 seconds marked by vertical red lines. From left to right, the columns represent neural responses to sucrose-paired ketone and terpene, control ketone and terpene, and airpuff-paired ketone and terpene (S_K, S_T, X_K, X_T, P_K, P_T). Data is pooled from 6 animals.

bioRxiv preprint doi: https://doi.org/10.1101/2023.08.01.551547; this version posted August 3, 2023. The copyright holder for this preprint (which was not certified by peer review) is the author/funder, who has granted bioRxiv a license to display the preprint in perpetuity. It is made available under aCC-BY 4.0 International license.

Figure S5:

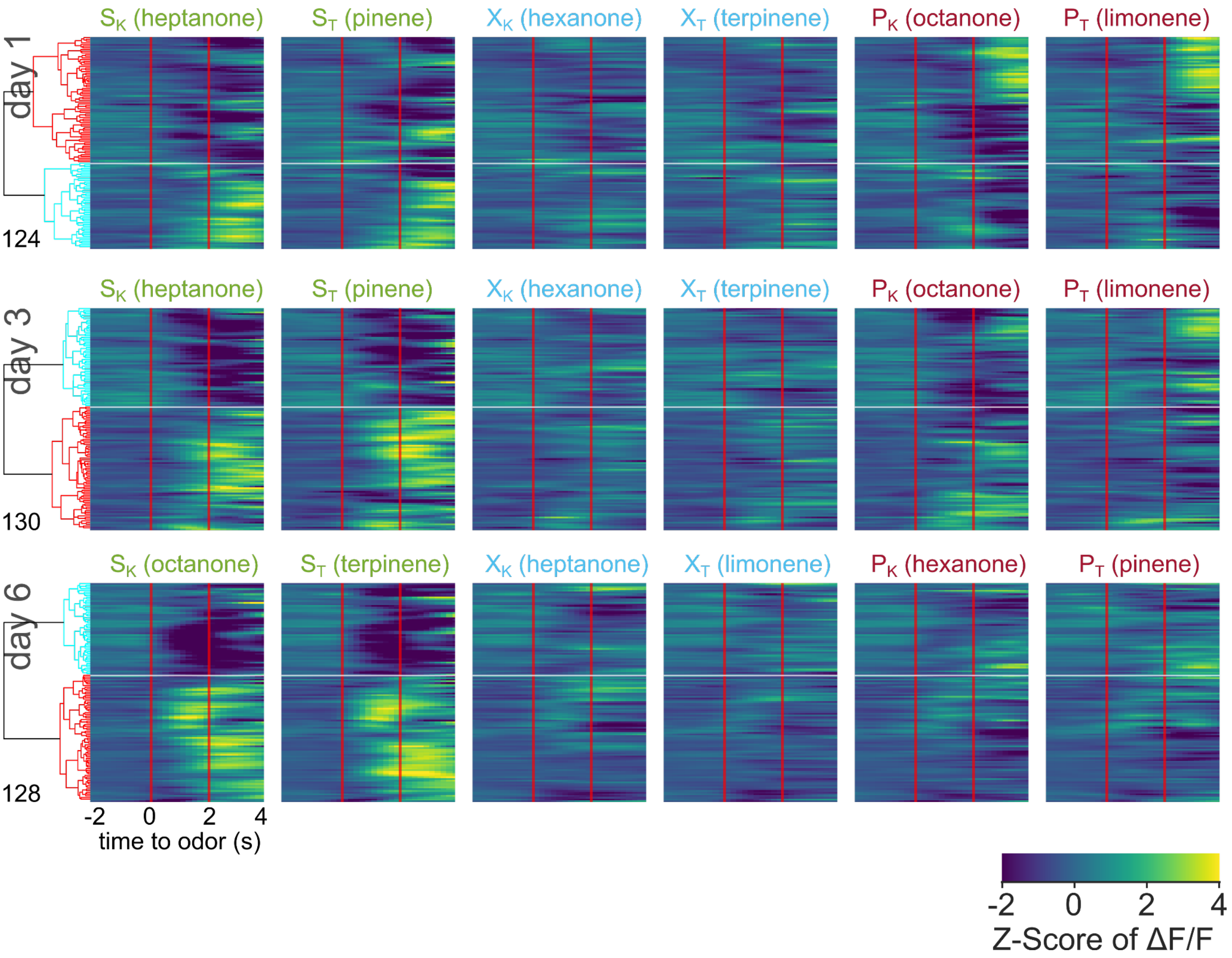

bioRxiv preprint doi: https://doi.org/10.1101/2023.08.01.551547; this version posted August 3, 2023. The copyright holder for this preprint (which was not certified by peer review) is the author/funder, who has granted bioRxiv a license to display the preprint in perpetuity. It is made available under aCC-BY 4.0 International license.

Figure S5. Pooled averaged-over-trials neural activity of all neurons from VP animals across days. Heatmap of odor-evoked activity in VP neurons from day 1, day 3, and day 6 of imaging. The fluorescence measurements from each neuron were averaged over trials, Z-scored, then pooled for hierarchical clustering. Neurons are grouped by similarity, with the dendrogram shown on the right. Horizontal white lines demarcate the boundaries between the 6 clusters. Odor delivered at 0-2 seconds marked by vertical red lines. From left to right, the columns represent neural responses to sucrose-paired ketone and terpene, control ketone and terpene, and airpuff-paired ketone and terpene (S_K, S_T, X_K, X_T, P_K, P_T). Data is pooled from 5 animals.

bioRxiv preprint doi: https://doi.org/10.1101/2023.08.01.551547; this version posted August 3, 2023. The copyright holder for this preprint (which was not certified by peer review) is the author/funder, who has granted bioRxiv a license to display the preprint in perpetuity. It is made available under aCC-BY 4.0 International license.

Figure S6:

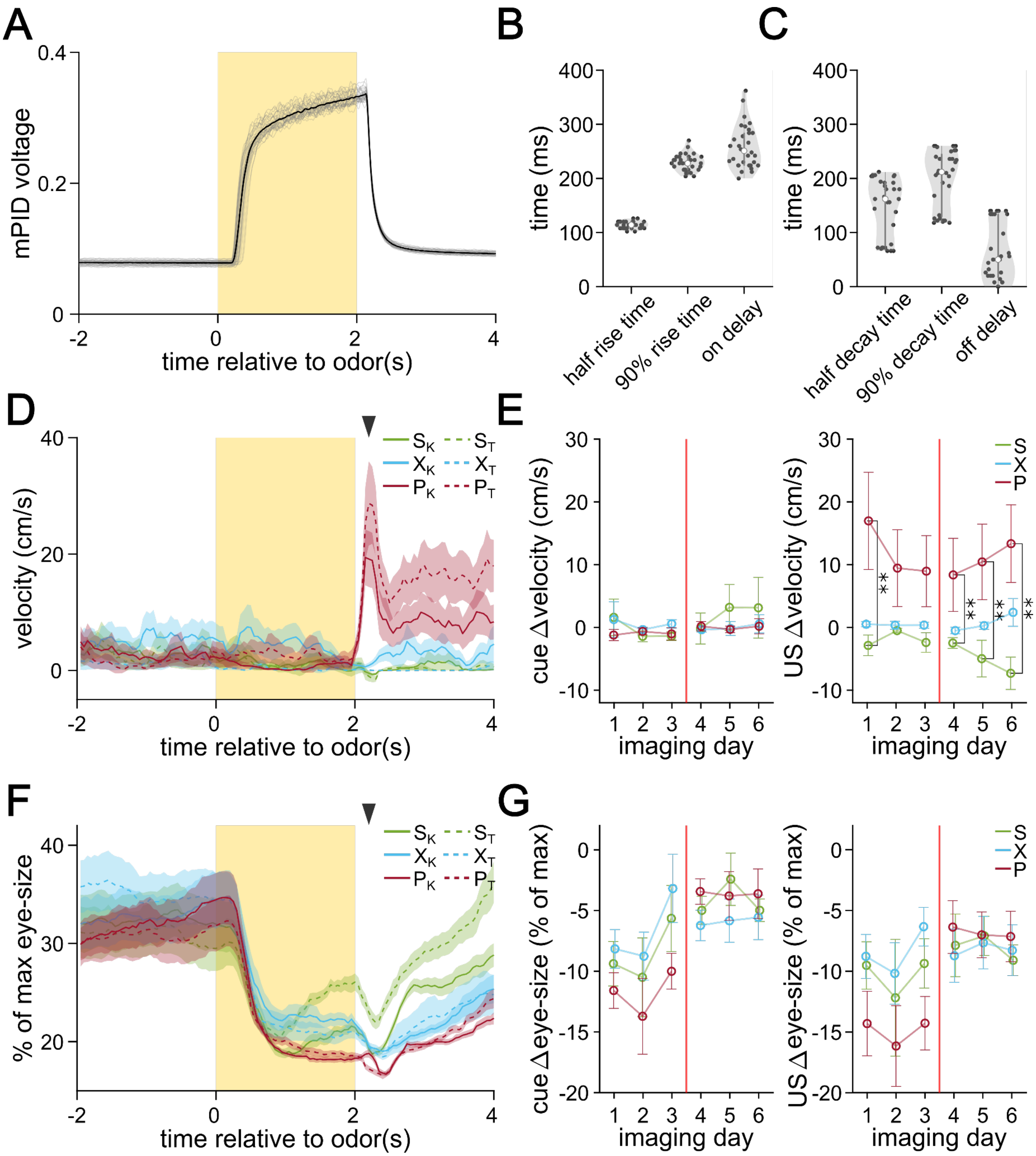

bioRxiv preprint doi: https://doi.org/10.1101/2023.08.01.551547; this version posted August 3, 2023. The copyright holder for this preprint (which was not certified by peer review) is the author/funder, who has granted bioRxiv a license to display the preprint in perpetuity. It is made available under aCC-BY 4.0 International license.

Figure S6. Extended behavioral analysis from imaging period. (**A**) mPID voltage reading in response to 30 trials of odor (terpinene) delivery. The time period during which the odor valve was turned on is shown by the yellow rectangle. Individual recordings are shown in gray and the average is shown in black. (**B**) On-kinetics of odor delivery. On delay refers to the interval between the valve turning on and the mPID voltage increasing by more than 10% of baseline. (**C**) Off-kinetics of odor delivery. Off delay refers to the interval between the odor valve turning off and the mPID voltage decreasing by more than 10% of its maximum. (**D**) Representative velocity of the head-fixed mouse in response to the 6 different odors measured by a digital encoder. The lines represent the average across 30 trials and the shaded areas represent the SEM. The black arrowhead marks when the US is delivered. (**E**) The difference in walking velocity in response to odor (left) and US delivery (right). Differences are calculated between the last second before odor delivery and the last second before the odor exposure (left) or the first half second after US delivery (right) grouped by US pairing. Circles represent the average across animals and the error bars show SEM. (**F**) Representative changes in range-normalized eye-size in response to the 6 different odors. (**G**) The difference in eye-size in response to odor (left) and US delivery (right). Differences are calculated between the last second before odor delivery and the last second before the odor exposure (left) or the first half second after US delivery (right) grouped by US pairing. Circles represent the average across animals and the error bars show SEM.

bioRxiv preprint doi: https://doi.org/10.1101/2023.08.01.551547; this version posted August 3, 2023. The copyright holder for this preprint (which was not certified by peer review) is the author/funder, who has granted bioRxiv a license to display the preprint in perpetuity. It is made available under aCC-BY 4.0 International license.

Figure S7:

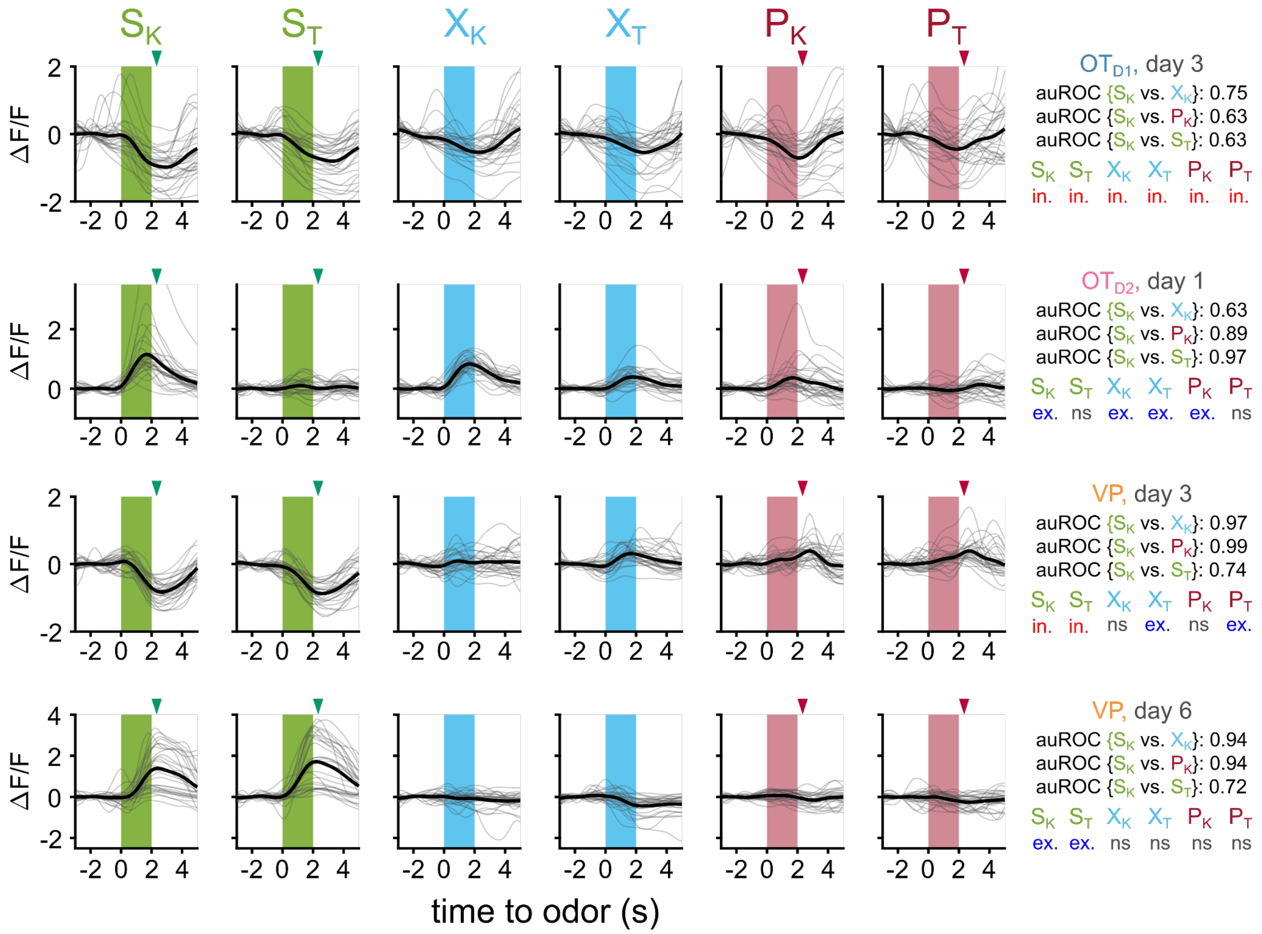

bioRxiv preprint doi: https://doi.org/10.1101/2023.08.01.551547; this version posted August 3, 2023. The copyright holder for this preprint (which was not certified by peer review) is the author/funder, who has granted bioRxiv a license to display the preprint in perpetuity. It is made available under aCC-BY 4.0 International license.

Figure S7. Traces of example neurons and their corresponding metrics. (**A**) Example traces from an OT_{D1} neuron recorded on day 3. Each column shows this neuron's response to a given odor across 30 trials (gray). The average across all trials is shown in black. For green and red arrowheads mark the time at which sucrose and airpuff were delivered, respectively. auROC values from single-neuron binary classifiers for discriminating {S_K vs. X_K}, {S_K vs. P_K}, and {S_K vs. S_T} are displayed on the right. Additionally, the results of statistical analysis to determine if this neuron reliably responded to each odor (in. = significant inhibitory response, exc. = significant excitatory response, ns= no significant difference between baseline and odor period). (**B**) Example traces from an OT_{D2} neuron recorded on day 1. (**C**) Example traces from a VP neuron recorded on day 3. (**D**) Example traces from a VP neuron recorded on day 6.

bioRxiv preprint doi: https://doi.org/10.1101/2023.08.01.551547; this version posted August 3, 2023. The copyright holder for this preprint (which was not certified by peer review) is the author/funder, who has granted bioRxiv a license to display the preprint in perpetuity. It is made available under aCC-BY 4.0 International license.

Figure S8:

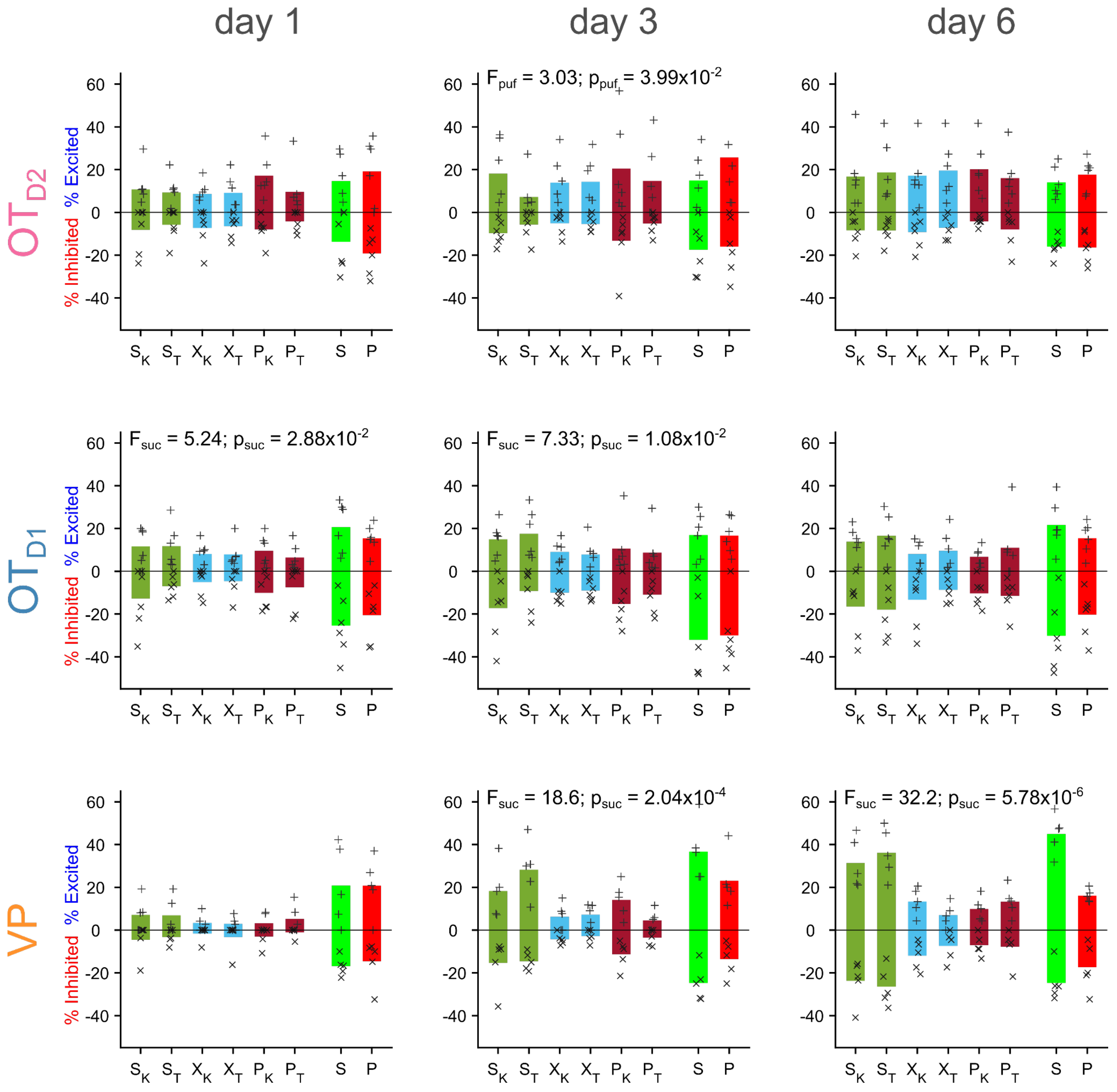

bioRxiv preprint doi: https://doi.org/10.1101/2023.08.01.551547; this version posted August 3, 2023. The copyright holder for this preprint (which was not certified by peer review) is the author/funder, who has granted bioRxiv a license to display the preprint in perpetuity. It is made available under aCC-BY 4.0 International license.

Figure S8. Percentage of neurons responsive to each odor across days. Bar graphs showing percentage of neurons from OT_{D2} on day 1 that were significantly excited or inhibited by each odor and each US (S for sucrose, P for airpuff). The average is shown by the bar while the + and x markers show individual biological replicates. For each region and imaging day pair, 3 one-way ANOVA's were run to test for the effect of sucrose, airpuff, or ketone vs. terpene on percentage responsiveness. Only the significant F-statistics are reported with their corresponding p-value.

bioRxiv preprint doi: https://doi.org/10.1101/2023.08.01.551547; this version posted August 3, 2023. The copyright holder for this preprint (which was not certified by peer review) is the author/funder, who has granted bioRxiv a license to display the preprint in perpetuity. It is made available under aCC-BY 4.0 International license.

Figure S9:

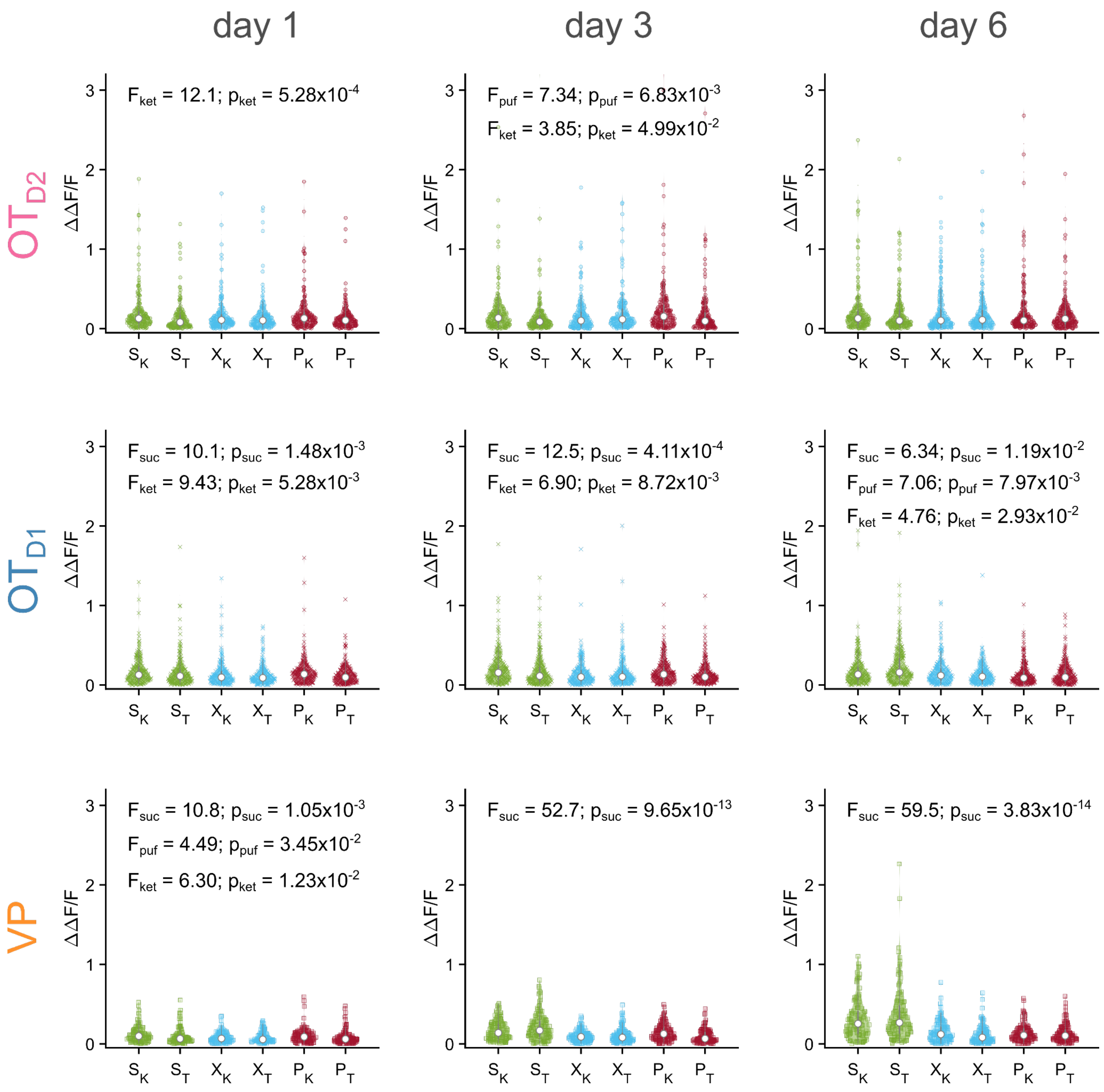

bioRxiv preprint doi: https://doi.org/10.1101/2023.08.01.551547; this version posted August 3, 2023. The copyright holder for this preprint (which was not certified by peer review) is the author/funder, who has granted bioRxiv a license to display the preprint in perpetuity. It is made available under aCC-BY 4.0 International license.

Figure S9. Distribution of response magnitudes to each odor across days. Violin plots showing the averaged-over-trials response magnitudes to each odor during the last second of odor exposure. For each region and imaging day pair, 3 one-way ANOVA's were performed to test for the effect of sucrose, airpuff, or ketone vs. terpene on response magnitude. Only the significant F-statistics are reported with their corresponding p-value.

bioRxiv preprint doi: https://doi.org/10.1101/2023.08.01.551547; this version posted August 3, 2023. The copyright holder for this preprint (which was not certified by peer review) is the author/funder, who has granted bioRxiv a license to display the preprint in perpetuity. It is made available under aCC-BY 4.0 International license.

Figure S10:

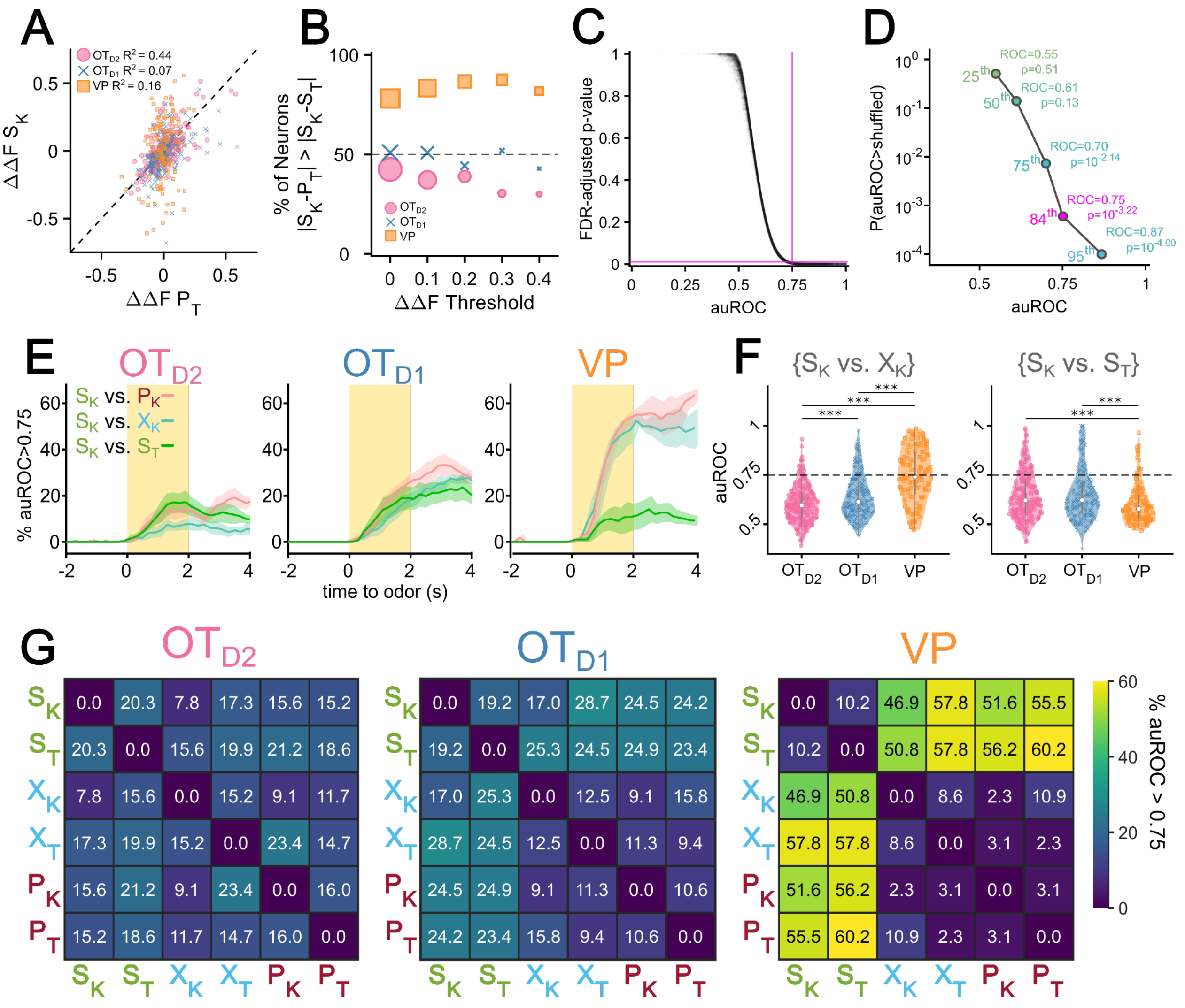

bioRxiv preprint doi: https://doi.org/10.1101/2023.08.01.551547; this version posted August 3, 2023. The copyright holder for this preprint (which was not certified by peer review) is the author/funder, who has granted bioRxiv a license to display the preprint in perpetuity. It is made available under aCC-BY 4.0 International license.

Figure S10. Pairwise analysis of single neuron odor encoding.

(**A**) Scatterplot comparing the magnitudes of S_K responses ($\Delta\Delta S_K$) to P_T responses ($\Delta\Delta P_T$). The dotted line represents the hypothetical scenario where $\Delta\Delta S_K = \Delta\Delta P_T$. For each population, the R^2 value of the 2-d distribution compared to the $\Delta\Delta S_K = \Delta\Delta P_T$ line is reported. (**B**) The percentage of neurons from each population where the difference between $\Delta\Delta S_K$ and $\Delta\Delta P_T$ is lower than that between $\Delta\Delta S_K$ and $\Delta\Delta S_T$. (**C**) Bootstrapped FDR-adjusted p-values as a function of auROC values of single-neuron binary classifiers. In total, there are 27,900 single-neuron binary classifiers (15 pairwise classifiers for each of the 1860 recordings across 3 regions and days 1, 3 and 6 of imaging). Each classifier was compared against 10,000 shuffles. Horizontal magenta line marks FDR-adjusted p-value of 0.001 and the vertical magenta line marks auROC of 0.75. (**D**) The 25th, 50th, 75th, 84th and 95th percentiles of auROC values and their corresponding unadjusted p-values. For auROC values that were greater than all 10,000 shuffles, a conservative p-value of 0.0001 was assigned. (**E**) The percentage of day 6 {S_K vs P_K}, {S_K vs X_K}, and {S_K vs S_T} auROC values greater than 0.75 as a function of time relative to odor, grouped by region. Lines represent the average across biological replicates and the shaded area shows the SEM. (**F**) Violin plot showing the distribution of pooled day 6 {S_K vs X_K} (left) and {S_K vs S_T} (right) auROC values grouped by region. Horizontal dotted line marks auROC = 0.75. (**G**) Heatmap of percentage of single-neuron pairwise classifiers with auROC>0.75. Classifiers were trained from neural activity recorded during the last second of odor exposure. Percent responsiveness was averaged across animals and grouped by region.

bioRxiv preprint doi: https://doi.org/10.1101/2023.08.01.551547; this version posted August 3, 2023. The copyright holder for this preprint (which was not certified by peer review) is the author/funder, who has granted bioRxiv a license to display the preprint in perpetuity. It is made available under aCC-BY 4.0 International license.

Figure S11:

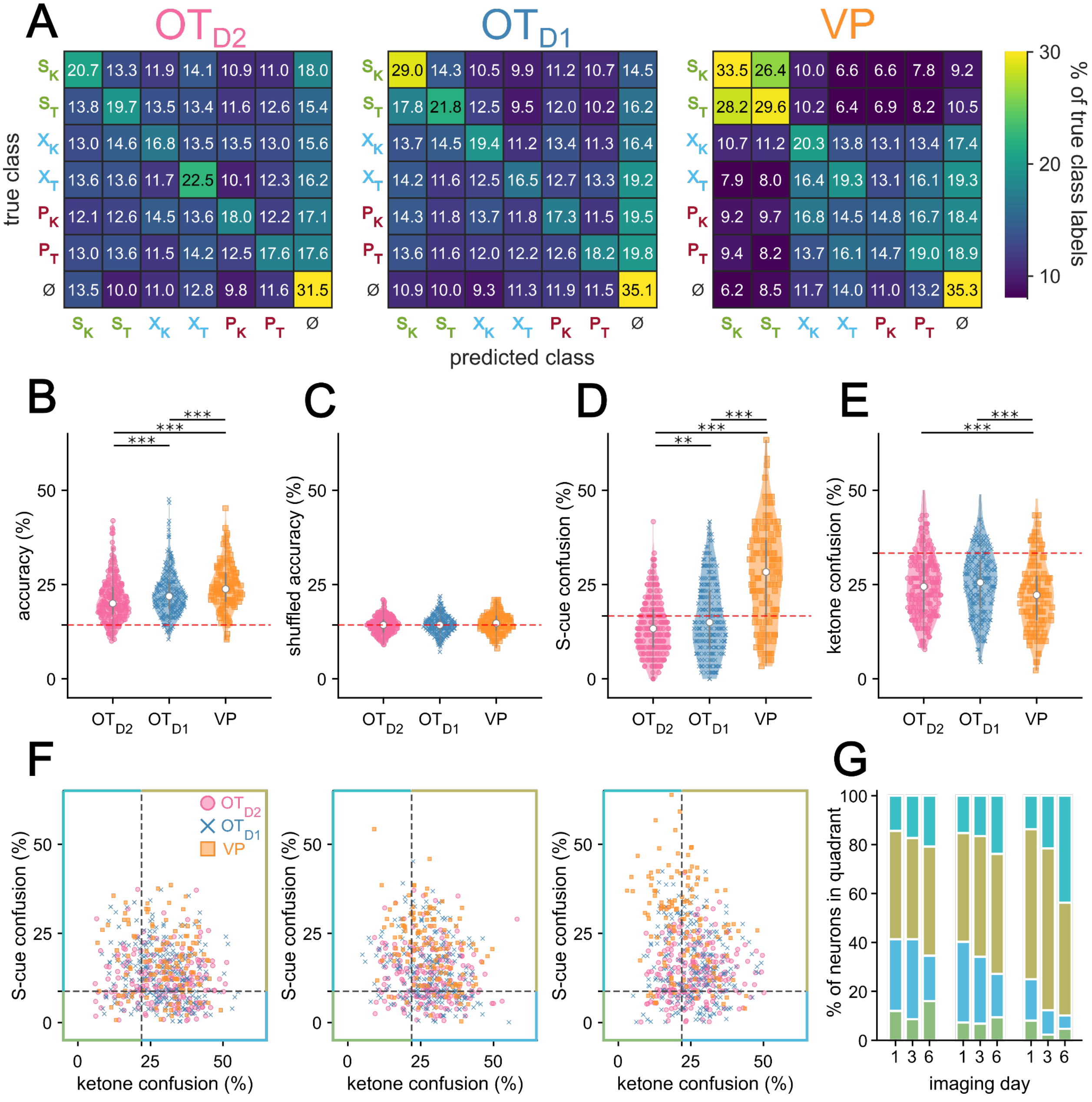

bioRxiv preprint doi: https://doi.org/10.1101/2023.08.01.551547; this version posted August 3, 2023. The copyright holder for this preprint (which was not certified by peer review) is the author/funder, who has granted bioRxiv a license to display the preprint in perpetuity. It is made available under aCC-BY 4.0 International license.

Figure S11. Multinomial analysis of single neuron odor encoding (**A**) Confusion matrix of single-neuron MNR classifiers trained on neural activity during the last second of odor exposure on day 6 of imaging. Rows represent the true class while columns represent the predicted class. Each confusion matrix is averaged across 10 k-fold and across all neurons of a given region. ϕ represents data taken from 30 pre-odor bins randomly sampled from -1.5 to -0.5 seconds relative to odor delivery. (**B**) Violin plot of single-neuron MNR classifier accuracy, averaged across 10 k-fold, grouped by region. (**C**) Violin plot of the single-neuron MNR classifier accuracy trained on shuffled data. Each data point represents the average across 10 shuffles. (**D**) Violin plot of MNR S-cue confusion, i.e. confusion between S_K and S_T. This corresponds to 1) when the true class was S_K but predicted class was S_T and 2) when the true class was S_T but the predicted class was S_K. (**E**) Violin plot of MNR confusion among all ketones. This corresponds when the true class was a ketone and the predicted class was a different ketone (e.g. true class = X_K and predicted class = P_K). (**F**) Scatterplot of each neuron's ketone confusion on the x-axis and S-cue confusion on the y-axis on days 1, 3, and 6 of imaging. (**G**) Stacked bar \graph showing the distribution of neurons from each population that fall into each of the 4 quadrants across the 3 different imaging days.

bioRxiv preprint doi: https://doi.org/10.1101/2023.08.01.551547; this version posted August 3, 2023. The copyright holder for this preprint (which was not certified by peer review) is the author/funder, who has granted bioRxiv a license to display the preprint in perpetuity. It is made available under aCC-BY 4.0 International license.

Figure S12:

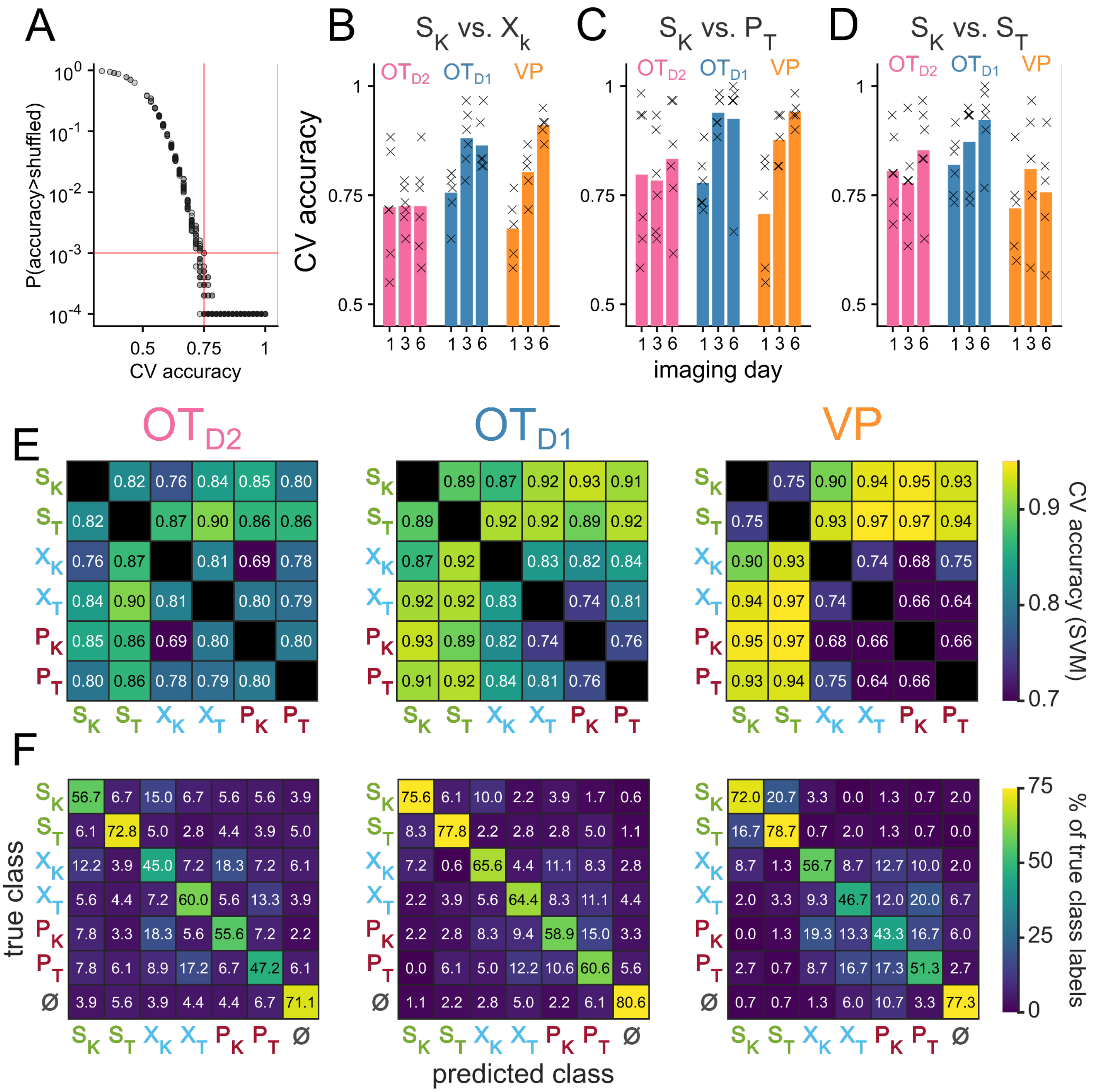

bioRxiv preprint doi: https://doi.org/10.1101/2023.08.01.551547; this version posted August 3, 2023. The copyright holder for this preprint (which was not certified by peer review) is the author/funder, who has granted bioRxiv a license to display the preprint in perpetuity. It is made available under aCC-BY 4.0 International license.

Figure S12. Analysis of population-level odor encoding (**A**) Scatterplot of CV-accuracy of linear classifiers trained on simultaneously-recorded neurons on the x-axis and their bootstrapped unadjusted p-values on the y-axis. Red horizontal line marks p = 0.001 and red red vertical line marks CV accuracy = 0.75. All classifiers with CV accuracy higher than 0.75 had p<0.001. In total, there are 765 binary classifiers (15 pairwise classifiers for each of the 51 recordings across 3 regions and days 1, 3, and 6 of imaging). Each classifier was compared against 10,000 shuffles. For auROC values that were greater than all 10,000 shuffles, a conservative p-value of 0.0001 was assigned. (**B**) The CV accuracy for {S_K vs X_K} binary classification trained on the last second of population-level activity. The bars represent the average across biological replicates. CV accuracy from individual animals are shown as x's. (**C**) Same as (B) but for {S_K vs. P_T} classification. (**D**) Same as (B) but for {S_K vs. S_T} classification. (**E**) Heatmap of CV accuracy from binary SVM's trained on day 6 of imaging with a radial basis function kernel. CV accuracy was averaged across biological replicates. (**F**) Confusion matrix of population-level MNR classifiers trained on neural activity during the last second of odor exposure on day 6 of imaging. Rows represent the true class while columns represent the predicted class. Each confusion matrix is averaged across biological replicates. ϕ represents data taken from 30 pre-odor bins randomly sampled from -1.5 to -0.5 seconds relative to odor delivery.

bioRxiv preprint doi: https://doi.org/10.1101/2023.08.01.551547; this version posted August 3, 2023. The copyright holder for this preprint (which was not certified by peer review) is the author/funder, who has granted bioRxiv a license to display the preprint in perpetuity. It is made available under aCC-BY 4.0 International license.

Figure S13:

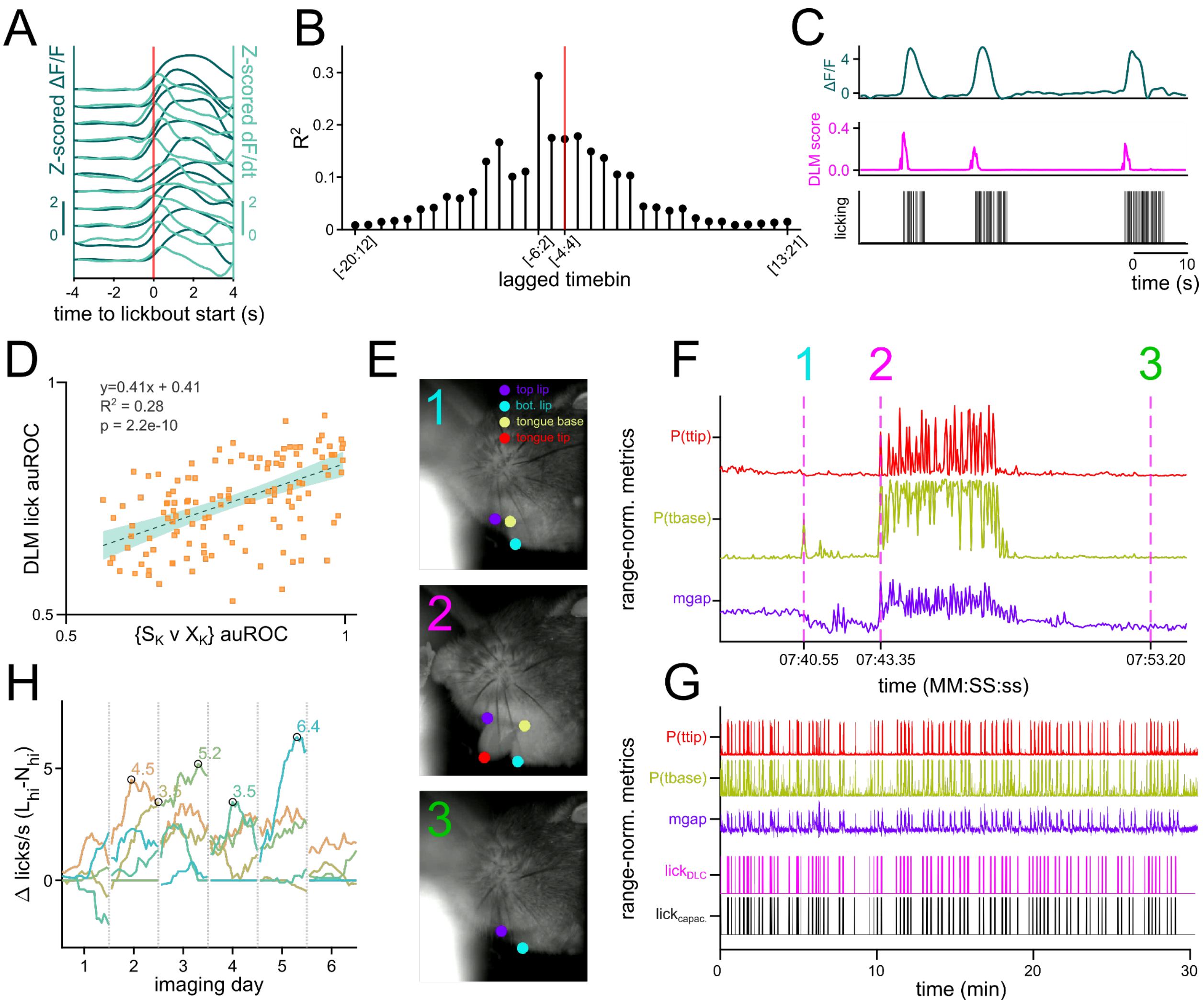

bioRxiv preprint doi: https://doi.org/10.1101/2023.08.01.551547; this version posted August 3, 2023. The copyright holder for this preprint (which was not certified by peer review) is the author/funder, who has granted bioRxiv a license to display the preprint in perpetuity. It is made available under aCC-BY 4.0 International license.

Figure S13. Camera-based detection of licking in head-fixed animals (**A-C**) Metrics of a representative neurons with activity that predicts licking. (**A**) Representative neuron's Z-scored ΔF/F (dark green) and Z-scored dF/dt (light green) aligned to the onset of a lickbout. Each line represents the same neuron's activity during an individual lickbout. (**B**) Stemplot showing an example of the lagged correlation between the onset of licking and the fluorescence of a neuron across 9 frames (1 frame = 0.2s). Time bins of various lags are shown on the x-axis (negative number denotes frames that precede onset of licking) and the resulting R^2 is plotted on the y-axis. Red vertical line marks the case where the 9 frames are centered on the onset of licking. As an example, [-6:2] refers to fluorescence between 1.2 seconds prior to lickbout onset and 0.4 seconds after lickbout onset. (**C**) The output of a distributed lag model (DLM) that predicts the onset of a lickbout from ΔF/F of a single neuron. ΔF/F (dark green, top), DLM score (magenta, middle), and the licking recorded by a capacitive sensor (black, bottom) are shown in parallel. The DLM model was trained using 9 distributed frames ([-6:2]) of ΔF/F for each frame of lickbout onset. (**D**) Scatterplot of day 6 VP neurons' DLM lick classifier auROC on the y-axis plotted against their mean {S_K vs. X_K} auROC on the x-axis. The slope, intercept, R^2, and p-value of the slope are shown on the top left corner. (**E**) 3 example snapshots of the camera feed during moving lick spout paradigm with overlay of DeepLabCut labeling. The coordinates of top lip, bottom lip, base of tongue (tbase), and tip of tongue (ttip) are displayed with a probability cutoff of 0.4. (**F**) Range-normalized metrics from DLC labeling. P(ttip) (red, top) is the probability score assigned to the labeling of the tongue tip. P(tbase) (yellow, middle) is the probability score assigned to the labeling of the tongue base. Mgap (purple, bottom) is the Euclidean distance between the top lip and the bottom lip. The 3 vertical magenta lines represent the timing of the 3 snapshots shown in (E). (**G**) The same range-normalized metrics as in (F) plotted against the ground truth licking data from capacitive sensor (black, bottom) and DLC-based licking classifier score (magenta, second from bottom). (**H**) Lineplot showing the difference in total licking to L_{hi} nd N_{hi} during the time bin (1.5-2.5 seconds after odor onset) used for most analyses plotted against imaging day for individual animals. The time of peak difference is circled in black.
